# Procedure via cross-Kerr nonlinearities for encoding single logical qubit information onto four-photon decoherence-free states

**DOI:** 10.1038/s41598-021-89809-w

**Published:** 2021-05-17

**Authors:** Jino Heo, Seong-Gon Choi

**Affiliations:** 1 Research Institute for Computer and Information Communication (RICIC), Chungbuk National University, Chungdae-ro 1, Seowon-Gu, Cheongju, Republic of Korea; 2 College of Electrical and Computer Engineering, Chungbuk National University, Chungdae-ro 1, Seowon-Gu, Cheongju, Republic of Korea

**Keywords:** Quantum optics, Information theory and computation, Quantum physics

## Abstract

We propose a photonic procedure using cross-Kerr nonlinearities (XKNLs) to encode single logical qubit information onto four-photon decoherence-free states. In quantum information processing, a decoherence-free subspace can secure quantum information against collective decoherence. Therefore, we design a procedure employing nonlinear optical gates, which are composed of XKNLs, quantum bus beams, and photon-number-resolving measurements with linear optical devices, to conserve quantum information by encoding quantum information onto four-photon decoherence-free states (single logical qubit information). Based on our analysis in quantifying the affection (photon loss and dephasing) of the decoherence effect, we demonstrate the experimental condition to acquire the reliable procedure of single logical qubit information having the robustness against the decoherence effect.

## Introduction

The influence of decoherence, nonunitary process, is one of the most significant obstacles hindering the reliable performance of various quantum information processing schemes, such as quantum communication^1–8^, quantum entanglement^9–14^, and quantum computation^15–22^. Therefore, the influence of decoherence should be reduced via active processes (quantum error corrections^23–25^, entanglement purifications^26–28^, and entanglement concentrations^30,31^) or passive processes (decoherence-free subspaces^32–36^).

In particular, utilizing a decoherence-free subspace prevents collective decoherence^32–36^ which occurs the identical decoherence occurring in each qubit in a system to be spread from one subspace to another subspace in a system when uncontrolled interactions between a system and environment affect the schemes of quantum information processing. Applications (passive processes)^37–48^ employing a decoherence-free subspace can provide immunity against collective decoherence^32–34^. For the passive process, a simple method is to encode quantum information onto two-qubit systems as a singlet state^36^ or three-qubit systems as an entangled *W* state^12,14,49,50^, and a three-qubit decoherence-free state^37–41, 51^. However, applications^12,14,36–41,49–51^ using two- or three-qubit systems can guarantee only a limited effect for maintaining the coherence of quantum information from the influence of collective decoherence in quantum channels. Hence, four-qubit decoherence-free subspaces, passive processes, utilizing various physical resources have been proposed to enhance the efficiency of coherent quantum information, e.g., linear optics with post-selections^41^, spontaneous parametric down conversions^52,53^, source of entangled state^54,55^, and cavity-QED^42,43,48^.

For the design of quantum information processing schemes, including passive processes, cross-Kerr nonlinearity (XKNL)^56–59^ is an appropriate candidate. Quantum controlled operations using XKNLs have been performed to implement various quantum information processing schemes by the indirect interaction between photons, signal systems, and probe beams, ancillary systems: coherent state, based on quantum non-demolition detections^10,12,14,16,18,56–64^. However, the decoherence effect (photon loss and dephasing)^57–59,63,65^, which results in the evolution from a quantum pure state to a mixed (classical) state, is inevitable when nonlinear optical gates via XKNLs are operated. To utilize quantum bus (qubus) beams and photon-number-resolving (PNR) measurements^10,12,14,16,18,66^ in nonlinear optical gates via XKNLs with a strong amplitude of coherent states (qubus beams), the decoherence effect should be reduced^57–59^.

In this study, we designed a photonic procedure based on nonlinear optical gates using XKNLs, qubus beams, and PNR measurements to encode quantum information onto four-photon decoherence-free states (single logical qubit information) to achieve robustness against collective decoherence^32–34^. Subsequently, using XKNLs, we quantified the efficiencies and performances of nonlinear optical gates under the decoherence effect (photon loss and dephasing^57–59,63,65^). In addition, we derived an experimental condition to reduce the decoherence effect in nonlinear optical gates.

We demonstrate that the proposed procedure for generating single logical qubit information (quantum information on four-photon decoherence-free states) with immunity against collective decoherence can be realized experimentally and that it is robust against the decoherence effect (photon loss and dephasing).

## Optical procedure via XKNLs for single logical qubit information

### Four-qubit decoherence-free state

To prevent quantum information in qubits from being affected by collective decoherence^32–34^, logical qubits using decoherence-free subspaces^37–48^ have been utilized. Herein, logical qubits {$\left|{0}_{\mathrm{L}}\rangle \right.$, $\left|{1}_{\mathrm{L}}\rangle \right.$} based on the four-qubit decoherence-free state are expressed as

1 $$\begin{aligned} {\left|{0}_{\mathrm{L}}\rangle \right.}_{1234} & \equiv \frac{1}{2}{\left(\left|0101\rangle \right.+\left|1010\rangle \right.-\left|0110\rangle \right.-\left|1001\rangle \right.\right)}_{1234}=\frac{1}{\sqrt{2}}{\left(\left|01\rangle \right.-\left|10\rangle \right.\right)}_{12} \otimes \frac{1}{\sqrt{2}}{\left(\left|01\rangle \right.-\left|10\rangle \right.\right)}_{34}, \\ {\left|{1}_{\mathrm{L}}\rangle \right.}_{1234} & \equiv \frac{1}{\sqrt{12}}{\left(2\left|0011\rangle \right.+2\left|1100\rangle \right.-\left|0101\rangle \right.-\left|1010\rangle \right.-\left|0110\rangle \right.-\left|1001\rangle \right.\right)}_{1234} \\ & =\frac{1}{\sqrt{3}}\left[{\left(\left|0011\rangle \right.+\left|1100\rangle \right.\right)}_{1234}-\frac{1}{\sqrt{2}}{\left(\left|01\rangle \right.+\left|10\rangle \right.\right)}_{12} \otimes \frac{1}{\sqrt{2}}{\left(\left|01\rangle \right.+\left|10\rangle \right.\right)}_{34}\right].\end{aligned}$$

Using the logical qubits in Eq. (1) (four-qubit decoherence-free states), we can encode arbitrary quantum information to acquire immunity against collective decoherence, as $\left| {\phi_{{\text{L}}} } \rangle \right. = \alpha \left| {0_{{\text{L}}} } \rangle \right. + \beta \left| {1_{{\text{L}}} }\rangle \right.$ with $\left| \alpha \right|^{2} + \left| \beta \right|^{2} = 1$ (single logical qubit information).

### Interaction of XKNL

**Figure 1:**
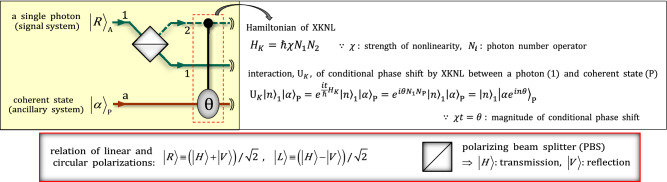
Schematic diagram of the interaction of XKNL: In the Figure, this interaction, conditional phase shift $\uptheta$ into the phase space of the coherent state P, ${\left|\alpha \rangle \right.}_{\mathrm{P}}$, is induced by the polarization ${\left|V\rangle \right.}_{\mathrm{P}}$ (path 2) of the photon A in the Kerr medium. Here, the photon and coherent state P play the roles of the control qubit, signal system ${\left|V\rangle \right.}_{\mathrm{P}}^{2}$, and target qubit, ancillary system ${\left|\alpha {e}^{i\theta }\rangle \right.}_{\mathrm{P}}^{\mathrm{a}}$, respectively.

For the interaction, ${\mathrm{U}}_{K}$, in Kerr medium, XKNL, which can be employed to realize the diverse quantum information processing schemes, the interaction between a photon A, signal system, and coherent state P, ancillary system, to induce a phase shift $\uptheta$ by the Kerr medium is described in Fig. 1. For example, we assume an input state, ${\left|R\rangle \right.}_{\mathrm{A}}{ \otimes \left|\alpha \rangle \right.}_{\mathrm{P}}$, to represent the interaction of the XKNL where $\left|\alpha \rangle \right.={e}^{-{\left|\alpha \right|}^{2}/2}\sum_{n=0}^{\infty }\frac{{\alpha }^{n}}{\sqrt{n!}}\left|n\rangle \right.$. After the input state passes through a polarizing beam splitter (PBS), the state is transformed by the interaction (dotted-red box in Fig. 1) of XKNL, as follows:

2 $${\left|R\rangle \right.}_{\mathrm{A}}^{1}{ \otimes \left|\alpha \rangle \right.}_{\mathrm{P}}^{\mathrm{a}} \stackrel{\mathrm{PBS}}{\to } \frac{1}{\sqrt{2}}\left({\left|H\rangle \right.}_{\mathrm{A}}^{1}+{\left|V\rangle \right.}_{\mathrm{A}}^{2}\right){ \otimes \left|\alpha \rangle \right.}_{\mathrm{P}}^{\mathrm{a}} \stackrel{{\mathrm{U}}_{K}}{\to } \frac{1}{\sqrt{2}}\left({\left|H\rangle \right.}_{\mathrm{A}}^{1}{ \otimes \left|\alpha \rangle \right.}_{\mathrm{P}}^{\mathrm{a}}+{{\mathrm{U}}_{K}\left|V\rangle \right.}_{\mathrm{A}}^{2}{ \otimes \left|\alpha \rangle \right.}_{\mathrm{P}}^{\mathrm{a}}\right)=\frac{1}{\sqrt{2}}\left({\left|H\rangle \right.}_{\mathrm{A}}^{1}{ \otimes \left|\alpha \rangle \right.}_{\mathrm{P}}^{\mathrm{a}}+{\left|V\rangle \right.}_{\mathrm{A}}^{2}{ \otimes \left|\alpha {e}^{i\theta }\rangle \right.}_{\mathrm{P}}^{\mathrm{a}}\right),$$

where the operations of the interaction, conditional phase shift, by XKNL and the PBS are described in Fig. 1. As described in Fig. 1 and Eq. (2), the phase of ancillary system, ${\left|\alpha \rangle \right.}_{\mathrm{P}}^{\mathrm{a}}$, is changed, according to the state, ${\left|H\rangle \right.}_{\mathrm{A}}^{1}$ or ${\left|V\rangle \right.}_{\mathrm{A}}^{2}$, of signal system via the interaction of XKNL. From this interaction, or procedure, we can realize quantum non-demolition detections^10,12,14,16,18,56–66^, which can obtain the information of signal system by the indirect detection in ancillary system, to utilize the various procedure for quantum information processing. For the interaction of XKNLs, the magnitude of XKNL can obtain to $\sim {10}^{-2}$, due to the electromagnetically induced transparency^67,68^. Recently, to the measurement-induced quantum operation on weak quantum states of light^69^ can generate a strong XKNL at the single photon level for the applicable quantum information processing. Also, for the Hamiltonian, ${H}_{K}=\hslash \chi {N}_{1}{N}_{2}$, of XKNL with $\chi =\left({g}_{1}^{2}{g}_{2}^{2}\right)/\left(\Delta {\Omega }_{c}^{2}\right)$, the scheme^70^ have been designed in circuit QED where ${g}_{i}$ is coupling strength, ${\Omega }_{c}$ is the transition strength between levels driven by a classical pump field with $\Delta$, detuning. This can show the Kerr medium can be substituted by circuit QED as the proposition^71^ in which the non-maximal entangled states of photons can be concentrated.

### Procedure via XKNLs for single logical qubit information

**Figure 2:**
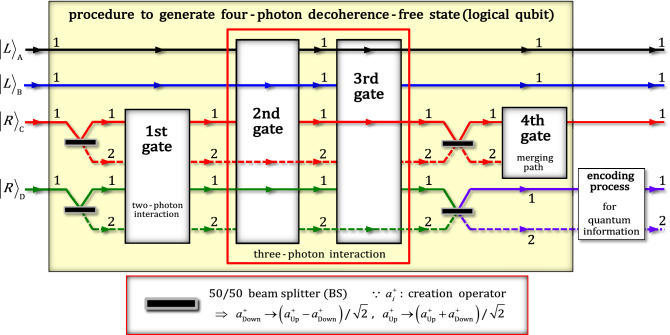
Procedure via XKNLs for single logical qubit information: First part is to generate the superposition of four-photon decoherence-free states. In this part, two-photon interaction of XKNLs is used in the first gate, and the fourth gate (via XKNLs) can merge photon paths. Meanwhile, the second and third gates are operated by three-photon interactions of XKNLs. In the second part, the encoding process can encode (arbitrary) quantum information onto four-photon decoherence-free states, which are output states of the first part.

Our procedure pertaining to single logical qubit information comprises two parts: in the first part, four-photon decoherence-free states (the superposition of logical qubits) are generated; in the second part, quantum information is encoded, as described in Fig. 2. In this procedure, all gates, first, second, third, fourth, and final gates, employ the interactions of XKNLs, qubus beams, and PNR measurements. For the single logical qubit information, we prepared the initial state as ${\left|\psi_{\mathrm{in}}\rangle \right.}_{\mathrm{ABCD}}^{1111}={\left|L\rangle \right.}_{\mathrm{A}}^{1} \otimes {\left|L\rangle \right.}_{\mathrm{B}}^{1} \otimes {\left|R\rangle \right.}_{\mathrm{C}}^{1} \otimes {\left|R\rangle \right.}_{\mathrm{D}}^{1}$, where $\left|R\rangle \right.$-right; $\left|L\rangle \right.$-left and linear $\left|H\rangle \right.$-horizontal; $\left|V\rangle \right.$-vertical represent the circular and linear polarizations of photon, respectively (Fig. 1). After the initial state, ${\left|{\psi }_{\mathrm{in}}\rangle \right.}_{\mathrm{ABCD}}^{1111}$ passes two 50/50 beam splitters (BSs), and the four-photon state ${\left|{\psi }_{0}\rangle \right.}_{\mathrm{ABCD}}$ can be expressed as

3 $${\left|\psi_{0}\rangle \right.}_{\mathrm{ABCD}}={\left|L\rangle \right.}_{\mathrm{A}}^{1} \otimes {\left|L\rangle \right.}_{\mathrm{B}}^{1} \otimes \left({\left|R\rangle \right.}_{\mathrm{C}}^{1}+{\left|R\rangle \right.}_{\mathrm{C}}^{2}\right)/\sqrt{2} \otimes \left({\left|R\rangle \right.}_{\mathrm{D}}^{1}+{\left|R\rangle \right.}_{\mathrm{D}}^{2}\right)/\sqrt{2},$$

where the operation of the BS is illustrated in Fig. 2.

**Figure 3:**
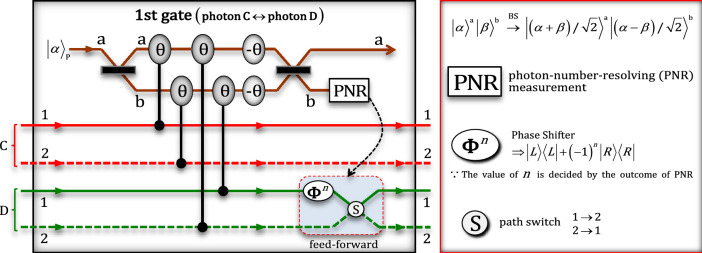
First gate (two-photon interactions between photons C and D) via XKNLs: For path arrangement of photons C and D, the first gate comprises XKNLs, qubus beams, PNR measurement, feed-forward, and linear optical devices. After PNR measurement, feed-forward (phase shifter and path switch) on photon D is either operated or not operated, depending on the result (photon number $n$) of PNR measurement.

In the first gate, two-photon interactions between photons C and D, shown in Fig. 3, four conditional phase shifts $\uptheta$ by XKNLs, two linear phase shifts $-\uptheta$, qubus beams (two BSs and PNR measurement), and feed-forward (phase shifter and path switch) were exploited for a controlled operation between photons C and D. After the state ${\left|\psi_{0}\rangle \right.}_{\mathrm{ABCD}}$ passes through the first gate, the pre-measurement (before PNR measurement) state $\left|\psi_{0}^{^\prime}\rangle \right._{\mathrm{ABCD}}$ can be expressed as

4 $${\left|\psi_{0}^{^{\prime}}\rangle \right.}_{\mathrm{ABCD}}={\left|L\rangle \right.}_{\mathrm{A}}^{1}{\left|L\rangle \right.}_{\mathrm{B}}^{1} \otimes \left[\frac{1}{\sqrt{2}}\left(\frac{1}{\sqrt{2}}{\left|R\rangle \right.}_{\mathrm{C}}^{1}{\left|R\rangle \right.}_{\mathrm{D}}^{1}+\frac{1}{\sqrt{2}}{\left|R\rangle \right.}_{\mathrm{C}}^{2}{\left|R\rangle \right.}_{\mathrm{D}}^{2}\right) \otimes {{\left|\alpha \rangle \right.}_{\mathrm{P}}^{\mathrm{a}}\left|0\rangle \right.}_{\mathrm{P}}^{\mathrm{b}}\right.+\left.\frac{1}{\sqrt{2}}{e}^{-\frac{{\left(\alpha \mathrm{sin}\theta \right)}^{2}}{2}}\sum_{n=0}^{\infty }\frac{{\left(i\alpha \mathrm{sin}\theta \right)}^{n}}{\sqrt{n!}}\left(\frac{1}{\sqrt{2}}{\left|R\rangle \right.}_{\mathrm{C}}^{1}{\left|R\rangle \right.}_{\mathrm{D}}^{2}+\frac{{\left(-1\right)}^{n}}{\sqrt{2}}{\left|R\rangle \right.}_{\mathrm{C}}^{2}{\left|R\rangle \right.}_{\mathrm{D}}^{1}\right) \otimes {\left|\alpha \mathrm{cos}\theta \rangle \right.}_{\mathrm{P}}^{\mathrm{a}}{\left|n\rangle \right.}_{\mathrm{P}}^{\mathrm{b}}\right],$$

where ${\left|\alpha \rangle \right.}_{\mathrm{P}}$ is the coherent state, probe beam: ancillary system. The operation of the BS in the qubus beam (coherent state) is shown in Fig. 3. ${\left|\pm i\alpha \mathrm{sin}\theta \rangle \right.}_{\mathrm{P}}={e}^{-{\left(\alpha \mathrm{sin}\theta \right)}^{2}/2}\sum_{n=0}^{\infty }\frac{{\left(\pm i\alpha \mathrm{sin}\theta \right)}^{n}}{\sqrt{n!}}{\left|n\rangle \right.}_{\mathrm{P}}$ for $\alpha \in {\mathbb{R}}$. Subsequently, by PNR measurement on path b of the qubus beams, if the outcome is $0$ (${\left|0\rangle \right.}_{\mathrm{P}}^{\mathrm{b}}$: no detection), then the output state, ${\left|{\psi }_{1}\rangle \right.}_{\mathrm{ABCD}}$ of the first gate can be obtained as ${\left|{\psi }_{1}\rangle \right.}_{\mathrm{ABCD}}={\left|L\rangle \right.}_{\mathrm{A}}^{1}{\left|L\rangle \right.}_{\mathrm{B}}^{1}\left({\left|R\rangle \right.}_{\mathrm{C}}^{1}{\left|R\rangle \right.}_{\mathrm{D}}^{1}+{\left|R\rangle \right.}_{\mathrm{C}}^{2}{\left|R\rangle \right.}_{\mathrm{D}}^{2}\right)/\sqrt{2}$ . Meanwhile, if the outcome is $n$ (${\left|n\rangle \right.}_{\mathrm{P}}^{\mathrm{b}}$: $n\ne 0$), then the output state ${\left|L\rangle \right.}_{\mathrm{A}}^{1}{\left|L\rangle \right.}_{\mathrm{B}}^{1}\left({\left|R\rangle \right.}_{\mathrm{C}}^{1}{\left|R\rangle \right.}_{\mathrm{D}}^{2}+{\left(-1\right)}^{n}{\left|R\rangle \right.}_{\mathrm{C}}^{2}{\left|R\rangle \right.}_{\mathrm{D}}^{1}\right)/\sqrt{2}$ can be transformed to state ${\left|{\psi }_{1}\rangle \right.}_{\mathrm{ABCD}}$ by feed-forward (phase shifter and path switch), as described in Fig. 3.

**Figure 4:**
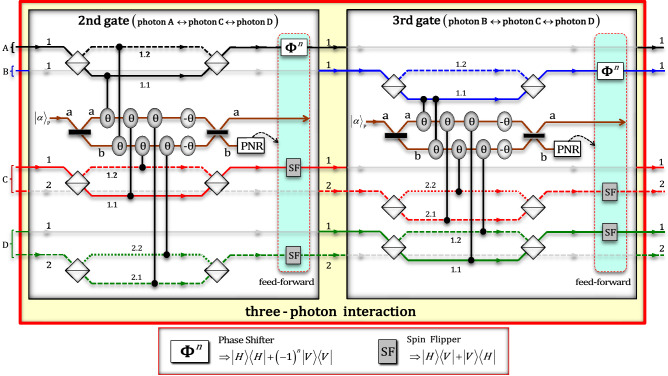
Second and third gates (three-photon interactions) via XKNLs: For controlled operations between three photons, two (second and third) gates consist of XKNLs, qubus beams, PNR measurement, feed-forward, and linear optical devices were used. Feed-forwards (phase shifters and spin flippers) of two gates are either operated or not operated on photons (**A**, **C**, and **D**: second gate) and (**B**, **C**, and **D**: third gate) depending on results of PNR measurements.

In the second and third gates, which interact with three-photon, shown in Fig. 4, conditional phase shifts $\uptheta$ by XKNLs, linear phase shifts $-\uptheta$, qubus beams (BSs and PNR measurements), feed-forwards (phase shifters and spin flippers), and linear optical devices, including polarizing beam splitters (PBSs), were utilized for controlled operations between three photons, i.e., (A, C, and D: second gate) and (B, C, and D: third gate). After the state ${\left|{\psi }_{1}\rangle \right.}_{\mathrm{ABCD}}$ (the output state of the first gate) passes through the second gate, the pre-measurement (before PNR measurement) state ${\left|\psi_{1}^{^{\prime}}\rangle \right.}_{\mathrm{ABCD}}$ can be expressed as

5 $$\begin{aligned} {\left|\psi_{1}^{^{\prime}}\rangle \right.}_{\mathrm{ABCD}} & =\frac{1}{\sqrt{2}}\left\{\frac{1}{2}{\left|H\rangle \right.}_{\mathrm{A}}^{1}{\left|L\rangle \right.}_{\mathrm{B}}^{1}\left({\left|V\rangle \right.}_{\mathrm{C}}^{1}{\left|R\rangle \right.}_{\mathrm{D}}^{1}+{\left|R\rangle \right.}_{\mathrm{C}}^{2}{\left|V\rangle \right.}_{\mathrm{D}}^{2}\right)-\frac{1}{2}{\left|V\rangle \right.}_{\mathrm{A}}^{1}{\left|L\rangle \right.}_{\mathrm{B}}^{1}\left({\left|H\rangle \right.}_{\mathrm{C}}^{1}{\left|R\rangle \right.}_{\mathrm{D}}^{1}+{\left|R\rangle \right.}_{\mathrm{C}}^{2}{\left|H\rangle \right.}_{\mathrm{D}}^{2}\right)\right\} \otimes {\left|\alpha \rangle \right.}_{\mathrm{P}}^{\mathrm{a}}{\left|0\rangle \right.}_{\mathrm{P}}^{\mathrm{b}} \\ & \quad +\frac{1}{\sqrt{2}}{e}^{-\frac{{\left(\alpha \mathrm{sin}\theta \right)}^{2}}{2}}\sum_{n=0}^{\infty }\frac{{\left(i\alpha \mathrm{sin}\theta \right)}^{n}}{\sqrt{n!}}\left\{\frac{1}{2}{\left|H\rangle \right.}_{\mathrm{A}}^{1}{\left|L\rangle \right.}_{\mathrm{B}}^{1}\left({\left|H\rangle \right.}_{\mathrm{C}}^{1}{\left|R\rangle \right.}_{\mathrm{D}}^{1}+{\left|R\rangle \right.}_{\mathrm{C}}^{2}{\left|H\rangle \right.}_{\mathrm{D}}^{2}\right)\right. \\ &\quad -\left.\frac{{\left(-1\right)}^{n}}{2}{\left|V\rangle \right.}_{\mathrm{A}}^{1}{\left|L\rangle \right.}_{\mathrm{B}}^{1}\left({\left|V\rangle \right.}_{\mathrm{C}}^{1}{\left|R\rangle \right.}_{\mathrm{D}}^{1}+{\left|R\rangle \right.}_{\mathrm{C}}^{2}{\left|V\rangle \right.}_{\mathrm{D}}^{2}\right)\right\} \otimes {\left|\alpha \mathrm{cos}\theta \rangle \right.}_{\mathrm{P}}^{\mathrm{a}}{\left|n\rangle \right.}_{\mathrm{P}}^{\mathrm{b}}. \end{aligned}$$

Depending on the PNR measurement result on path b of the qubus beams, if the outcome is $0$ (${\left|0\rangle \right.}_{\mathrm{P}}^{\mathrm{b}}$: no detection), then the output state, ${\left|{\psi }_{2}\rangle \right.}_{\mathrm{ABCD}}$ of the second gate can be obtained as ${\left|{\psi }_{2}\rangle \right.}_{\mathrm{ABCD}}=\left\{{\left|H\rangle \right.}_{\mathrm{A}}^{1}{\left|L\rangle \right.}_{\mathrm{B}}^{1}\left({\left|V\rangle \right.}_{\mathrm{C}}^{1}{\left|R\rangle \right.}_{\mathrm{D}}^{1}+{\left|R\rangle \right.}_{\mathrm{C}}^{2}{\left|V\rangle \right.}_{\mathrm{D}}^{2}\right)-{\left|V\rangle \right.}_{\mathrm{A}}^{1}{\left|L\rangle \right.}_{\mathrm{B}}^{1}\left({\left|H\rangle \right.}_{\mathrm{C}}^{1}{\left|R\rangle \right.}_{\mathrm{D}}^{1}+{\left|R\rangle \right.}_{\mathrm{C}}^{2}{\left|H\rangle \right.}_{\mathrm{D}}^{2}\right)\right\}/2$. Otherwise, if $n$ (${\left|n\rangle \right.}_{\mathrm{P}}^{\mathrm{b}}$: $n\ne 0$), then the output state can be changed to ${\left|{\psi }_{2}\rangle \right.}_{\mathrm{ABCD}}$ by feed-forwards (phase shifter and spin flippers). Subsequently, the state ${\left|{\psi }_{2}\rangle \right.}_{\mathrm{ABCD}}$ enters the third gate for another controlled operation. After the third gate, the state ${\left|\psi_{2}^{^{\prime}}\rangle \right.}_{\mathrm{ABCD}}$ (before PNR measurement) can be written as

6 $$\begin{aligned}{\left|\psi_{2}^{^{\prime}}\rangle \right.}_{\mathrm{ABCD}} & =\frac{1}{\sqrt{2}}\left[\frac{1}{2\sqrt{2}}\left\{{\left|H\rangle \right.}_{\mathrm{A}}^{1}{\left|H\rangle \right.}_{\mathrm{B}}^{1}\left({\left|V\rangle \right.}_{\mathrm{C}}^{1}{\left|V\rangle \right.}_{\mathrm{D}}^{1}+{\left|V\rangle \right.}_{\mathrm{C}}^{2}{\left|V\rangle \right.}_{\mathrm{D}}^{2}\right)-{\left|V\rangle \right.}_{\mathrm{A}}^{1}{\left|H\rangle \right.}_{\mathrm{B}}^{1}\left({\left|H\rangle \right.}_{\mathrm{C}}^{1}{\left|V\rangle \right.}_{\mathrm{D}}^{1}+{\left|V\rangle \right.}_{\mathrm{C}}^{2}{\left|H\rangle \right.}_{\mathrm{D}}^{2}\right)\right\}\right. \\ & \quad +\left.\frac{1}{2\sqrt{2}}\left\{{\left|V\rangle \right.}_{\mathrm{A}}^{1}{\left|V\rangle \right.}_{\mathrm{B}}^{1}\left({\left|H\rangle \right.}_{\mathrm{C}}^{1}{\left|H\rangle \right.}_{\mathrm{D}}^{1}+{\left|H\rangle \right.}_{\mathrm{C}}^{2}{\left|H\rangle \right.}_{\mathrm{D}}^{2}\right)-{\left|H\rangle \right.}_{\mathrm{A}}^{1}{\left|V\rangle \right.}_{\mathrm{B}}^{1}\left({\left|H\rangle \right.}_{\mathrm{C}}^{2}{\left|V\rangle \right.}_{\mathrm{D}}^{2}+{\left|V\rangle \right.}_{\mathrm{C}}^{1}{\left|H\rangle \right.}_{\mathrm{D}}^{1}\right)\right\}\right] \otimes {\left|\alpha \rangle \right.}_{\mathrm{P}}^{\mathrm{a}}{\left|0\rangle \right.}_{\mathrm{P}}^{\mathrm{b}} \\ & \quad +\frac{1}{\sqrt{2}}{e}^{-\frac{{\left(\alpha \mathrm{sin}\theta \right)}^{2}}{2}}\sum_{n=0}^{\infty }\frac{{\left(i\alpha \mathrm{sin}\theta \right)}^{n}}{\sqrt{n!}}\left[\frac{1}{2\sqrt{2}}\left\{{\left|H\rangle \right.}_{\mathrm{A}}^{1}{\left|H\rangle \right.}_{\mathrm{B}}^{1}\left({\left|V\rangle \right.}_{\mathrm{C}}^{1}{\left|H\rangle \right.}_{\mathrm{D}}^{1}+{\left|H\rangle \right.}_{\mathrm{C}}^{2}{\left|V\rangle \right.}_{\mathrm{D}}^{2}\right)-{\left|V\rangle \right.}_{\mathrm{A}}^{1}{\left|H\rangle \right.}_{\mathrm{B}}^{1}\left({\left|H\rangle \right.}_{\mathrm{C}}^{1}{\left|H\rangle \right.}_{\mathrm{D}}^{1}+{\left|H\rangle \right.}_{\mathrm{C}}^{2}{\left|H\rangle \right.}_{\mathrm{D}}^{2}\right)\right\}\right. \\ & \quad +\left.\frac{{\left(-1\right)}^{n}}{2\sqrt{2}}\left\{{\left|V\rangle \right.}_{\mathrm{A}}^{1}{\left|V\rangle \right.}_{\mathrm{B}}^{1}\left({\left|H\rangle \right.}_{\mathrm{C}}^{1}{\left|V\rangle \right.}_{\mathrm{D}}^{1}+{\left|V\rangle \right.}_{\mathrm{C}}^{2}{\left|H\rangle \right.}_{\mathrm{D}}^{2}\right)-{\left|H\rangle \right.}_{\mathrm{A}}^{1}{\left|V\rangle \right.}_{\mathrm{B}}^{1}\left({\left|V\rangle \right.}_{\mathrm{C}}^{1}{\left|V\rangle \right.}_{\mathrm{D}}^{1}+{\left|V\rangle \right.}_{\mathrm{C}}^{2}{\left|V\rangle \right.}_{\mathrm{D}}^{2}\right)\right\}\right] \otimes {\left|\alpha \mathrm{cos}\theta \rangle \right.}_{\mathrm{P}}^{\mathrm{a}}{\left|n\rangle \right.}_{\mathrm{P}}^{\mathrm{b}}. \end{aligned}$$

After the operations applying feed-forwards or not in Fig. 4, owing to the outcome of the PNR measurement, the output state ${\left|{\psi }_{3}\rangle \right.}_{\mathrm{ABCD}}$ of the third gate can be expressed as

7 $$\begin{aligned} {\left|{\psi }_{3}\rangle \right.}_{\mathrm{ABCD}} & =\frac{1}{2}\left[{\left|H\rangle \right.}_{\mathrm{A}}^{1}{\left|H\rangle \right.}_{\mathrm{B}}^{1} \otimes \left({\left|V\rangle \right.}_{\mathrm{C}}^{1}{\left|V\rangle \right.}_{\mathrm{D}}^{1}+{\left|V\rangle \right.}_{\mathrm{C}}^{2}{\left|V\rangle \right.}_{\mathrm{D}}^{2}\right)/\sqrt{2}-{\left|V\rangle \right.}_{\mathrm{A}}^{1}{\left|H\rangle \right.}_{\mathrm{B}}^{1} \otimes \left({\left|H\rangle \right.}_{\mathrm{C}}^{1}{\left|V\rangle \right.}_{\mathrm{D}}^{1}+{\left|V\rangle \right.}_{\mathrm{C}}^{2}{\left|H\rangle \right.}_{\mathrm{D}}^{2}\right)\right./\sqrt{2} \\ & \quad +\left.{\left|V\rangle \right.}_{\mathrm{A}}^{1}{\left|V\rangle \right.}_{\mathrm{B}}^{1} \otimes \left({\left|H\rangle \right.}_{\mathrm{C}}^{1}{\left|H\rangle \right.}_{\mathrm{D}}^{1}+{\left|H\rangle \right.}_{\mathrm{C}}^{2}{\left|H\rangle \right.}_{\mathrm{D}}^{2}\right)/\sqrt{2}-{\left|H\rangle \right.}_{\mathrm{A}}^{1}{\left|V\rangle \right.}_{\mathrm{B}}^{1} \otimes \left({\left|H\rangle \right.}_{\mathrm{C}}^{2}{\left|V\rangle \right.}_{\mathrm{D}}^{2}+{\left|V\rangle \right.}_{\mathrm{C}}^{1}{\left|H\rangle \right.}_{\mathrm{D}}^{1}\right)/\sqrt{2}\right]. \end{aligned}$$

Subsequently, as described in Fig. 2, two BSs were applied to photons C and D of the output state ${\left|{\psi }_{3}\rangle \right.}_{\mathrm{ABCD}}$ in Eq. 7. Next, the output state ${\left|{\psi }_{3}\rangle \right.}_{\mathrm{ABCD}}$ was transformed to state ${\left|\psi_{3}^{^{\prime}}\rangle \right.}_{\mathrm{ABCD}}$ of the superposed (four-photon) decoherence-free states, as follows:

8 $$\begin{aligned}{\left|{\psi }_{3}^{^{\prime}}\rangle \right.}_{\mathrm{ABCD}} & =\frac{1}{4\sqrt{2}}\left[\left\{{\left|H\rangle \right.}_{\mathrm{A}}^{1}{\left|V\rangle \right.}_{\mathrm{B}}^{1}{\left|H\rangle \right.}_{\mathrm{C}}^{1}{\left|V\rangle \right.}_{\mathrm{D}}^{2}+{\left|V\rangle \right.}_{\mathrm{A}}^{1}{\left|H\rangle \right.}_{\mathrm{B}}^{1}{\left|V\rangle \right.}_{\mathrm{C}}^{1}{\left|H\rangle \right.}_{\mathrm{D}}^{2}-{\left|H\rangle \right.}_{\mathrm{A}}^{1}{\left|V\rangle \right.}_{\mathrm{B}}^{1}{\left|V\rangle \right.}_{\mathrm{C}}^{1}{\left|H\rangle \right.}_{\mathrm{D}}^{2}-{\left|V\rangle \right.}_{\mathrm{A}}^{1}{\left|H\rangle \right.}_{\mathrm{B}}^{1}{\left|H\rangle \right.}_{\mathrm{C}}^{1}{\left|V\rangle \right.}_{\mathrm{D}}^{2}\right\}\right. \\ & \quad +\left\{{\left|H\rangle \right.}_{\mathrm{A}}^{1}{\left|V\rangle \right.}_{\mathrm{B}}^{1}{\left|H\rangle \right.}_{\mathrm{C}}^{2}{\left|V\rangle \right.}_{\mathrm{D}}^{1}+{\left|V\rangle \right.}_{\mathrm{A}}^{1}{\left|H\rangle \right.}_{\mathrm{B}}^{1}{\left|V\rangle \right.}_{\mathrm{C}}^{2}{\left|H\rangle \right.}_{\mathrm{D}}^{1}-{\left|H\rangle \right.}_{\mathrm{A}}^{1}{\left|V\rangle \right.}_{\mathrm{B}}^{1}{\left|V\rangle \right.}_{\mathrm{C}}^{2}{\left|H\rangle \right.}_{\mathrm{D}}^{1}-{\left|V\rangle \right.}_{\mathrm{A}}^{1}{\left|H\rangle \right.}_{\mathrm{B}}^{1}{\left|H\rangle \right.}_{\mathrm{C}}^{2}{\left|V\rangle \right.}_{\mathrm{D}}^{1}\right\} \\ & \quad +\left\{2{\left|H\rangle \right.}_{\mathrm{A}}^{1}{\left|H\rangle \right.}_{\mathrm{B}}^{1}{\left|V\rangle \right.}_{\mathrm{C}}^{1}{\left|V\rangle \right.}_{\mathrm{D}}^{1}+2{\left|V\rangle \right.}_{\mathrm{A}}^{1}{\left|V\rangle \right.}_{\mathrm{B}}^{1}{\left|H\rangle \right.}_{\mathrm{C}}^{1}{\left|H\rangle \right.}_{\mathrm{D}}^{1}-{\left|V\rangle \right.}_{\mathrm{A}}^{1}{\left|H\rangle \right.}_{\mathrm{B}}^{1}{\left|H\rangle \right.}_{\mathrm{C}}^{1}{\left|V\rangle \right.}_{\mathrm{D}}^{1}-{\left|H\rangle \right.}_{\mathrm{A}}^{1}{\left|V\rangle \right.}_{\mathrm{B}}^{1}{\left|V\rangle \right.}_{\mathrm{C}}^{1}{\left|H\rangle \right.}_{\mathrm{D}}^{1}\right. \\ & \quad \left.-{\left|H\rangle \right.}_{\mathrm{A}}^{1}{\left|V\rangle \right.}_{\mathrm{B}}^{1}{\left|H\rangle \right.}_{\mathrm{C}}^{1}{\left|V\rangle \right.}_{\mathrm{D}}^{1}-{\left|V\rangle \right.}_{\mathrm{A}}^{1}{\left|H\rangle \right.}_{\mathrm{B}}^{1}{\left|V\rangle \right.}_{\mathrm{C}}^{1}{\left|H\rangle \right.}_{\mathrm{D}}^{1}\right\}+\left\{2{\left|H\rangle \right.}_{\mathrm{A}}^{1}{\left|H\rangle \right.}_{\mathrm{B}}^{1}{\left|V\rangle \right.}_{\mathrm{C}}^{2}{\left|V\rangle \right.}_{\mathrm{D}}^{2}+2{\left|V\rangle \right.}_{\mathrm{A}}^{1}{\left|V\rangle \right.}_{\mathrm{B}}^{1}{\left|H\rangle \right.}_{\mathrm{C}}^{2}{\left|H\rangle \right.}_{\mathrm{D}}^{2}\right. \\ & \quad \left.\left.-\left.{\left|V\rangle \right.}_{\mathrm{A}}^{1}{\left|H\rangle \right.}_{\mathrm{B}}^{1}{\left|H\rangle \right.}_{\mathrm{C}}^{2}{\left|V\rangle \right.}_{\mathrm{D}}^{2}-{\left|H\rangle \right.}_{\mathrm{A}}^{1}{\left|V\rangle \right.}_{\mathrm{B}}^{1}{\left|V\rangle \right.}_{\mathrm{C}}^{2}{\left|H\rangle \right.}_{\mathrm{D}}^{2}-{\left|H\rangle \right.}_{\mathrm{A}}^{1}{\left|V\rangle \right.}_{\mathrm{B}}^{1}{\left|H\rangle \right.}_{\mathrm{C}}^{2}{\left|V\rangle \right.}_{\mathrm{D}}^{2}-{\left|V\rangle \right.}_{\mathrm{A}}^{1}{\left|H\rangle \right.}_{\mathrm{B}}^{1}{\left|V\rangle \right.}_{\mathrm{C}}^{2}{\left|H\rangle \right.}_{\mathrm{D}}^{2}\right)\right\}\right] \\& \equiv \frac{1}{\sqrt{2}}\left(\frac{1}{2}{\left|{0}_{\mathrm{L}}\rangle \right.}_{\mathrm{ABCD}}^{1112}+\frac{\sqrt{3}}{2}{\left|{1}_{\mathrm{L}}\rangle \right.}_{\mathrm{ABCD}}^{1111}\right)+\frac{1}{\sqrt{2}}\left(\frac{1}{2}{\left|{0}_{\mathrm{L}}\rangle \right.}_{\mathrm{ABCD}}^{1121}+\frac{\sqrt{3}}{2}{\left|{1}_{\mathrm{L}}\rangle \right.}_{\mathrm{ABCD}}^{1122}\right), \end{aligned}$$

where we define the polarizations ($|H\rangle$ and $|V\rangle$) of photons corresponding to states ($|0\rangle$ and $|1\rangle$) of the qubit as $\left\{\left|H\rangle \right., \left|V\rangle \right.\right\}\equiv \left\{\left|0\rangle \right., \left|1\rangle \right.\right\}$. Hence, state ${|\psi_{3}^{^{\prime}}\rangle }_{\mathrm{ABCD}}$ is the photonic superposition of logical qubits (four-qubit decoherence-free states in Eq. 1), according to the paths of photons C and D.

**Figure 5:**
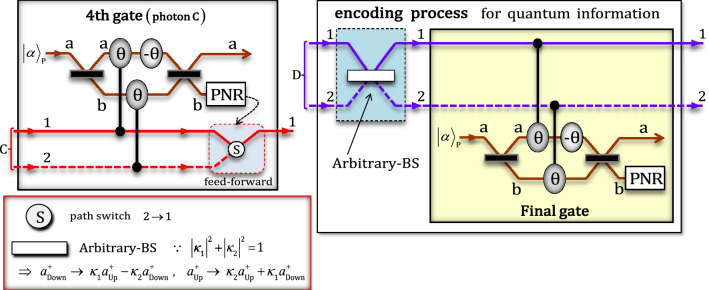
Fourth gate via XKNLs; encoding process with arbitrary-BS and final gate (XKNLs): The fourth gate merged the path of photon C using XKNLs, qubus beams, PNR measurement, and feed-forward (path switch). During encoding, the arbitrary-BS (linear optical device) and final gate (via XKNLs) encode arbitrary quantum information onto four-photon decoherence-free states (single logical qubit information).

In the fourth gate (photon C) shown in Fig. 5, two conditional phase shifts $\uptheta$ by XKNLs, one linear phase shift $-\uptheta$, qubus beams (two BSs and PNR measurement), and feed-forward (path switch) were utilized to merge the path of photon C (path 1 and path 2 → path 1). After state ${|\psi_{3}^{^{\prime}}\rangle}_{\mathrm{ABCD}}$ passes through the fourth gate, the pre-measurement (before PNR) state ${|\psi_{3}^{{^{\prime\prime}}}\rangle }_{\mathrm{ABCD}}$ can be expressed as

9 $$\begin{aligned} \left|\psi _{3} ^{{\prime \prime }}\right.\rangle _{{{\text{ABCD}}}} & = \frac{1}{{\sqrt 2 }}\left( {\frac{1}{2}\left| {0_{{\text{L}}} \rangle } \right._{{{\text{ABCD}}}}^{{1112}} + \frac{{\sqrt 3 }}{2}\left| {1_{{\text{L}}} \rangle } \right._{{{\text{ABCD}}}}^{{1111}} } \right) \otimes \left| {\alpha \rangle } \right._{{\text{P}}}^{{\text{a}}} \left| {0\rangle } \right._{{\text{P}}}^{{\text{b}}} \\ & \quad +\frac{1}{\sqrt{2}}{e}^{-\frac{{\left(\alpha \mathrm{sin}\theta \right)}^{2}}{2}}\sum_{n=0}^{\infty }\frac{{\left(-i\alpha \mathrm{sin}\theta \right)}^{n}}{\sqrt{n!}}\left(\frac{1}{2}{\left|{0}_{\mathrm{L}}\rangle \right.}_{\mathrm{ABCD}}^{1121}+\frac{\sqrt{3}}{2}{\left|{1}_{\mathrm{L}}\rangle \right.}_{\mathrm{ABCD}}^{1122}\right) \otimes {\left|\alpha \mathrm{cos}\theta \rangle \right.}_{\mathrm{P}}^{\mathrm{a}}{\left|n\rangle \right.}_{\mathrm{P}}^{\mathrm{b}}.\end{aligned}$$

Subsequently, by PNR measurement on path b of the qubus beams, if the outcome is $0$ (${\left|0\rangle \right.}_{\mathrm{P}}^{\mathrm{b}}$: no detection), then output state is ${\left|{\psi }_{4}\rangle \right.}_{\mathrm{ABCD}}=\left({\left|{0}_{\mathrm{L}}\rangle \right.}_{\mathrm{ABCD}}^{1112}+\sqrt{3}{\left|{1}_{\mathrm{L}}\rangle \right.}_{\mathrm{ABCD}}^{1111}\right)/2$. Meanwhile, if the outcome is $n$ (${\left|n\rangle \right.}_{\mathrm{P}}^{\mathrm{b}}$: $n\ne 0$), then the output state $\left({\left|{0}_{\mathrm{L}}\rangle \right.}_{\mathrm{ABCD}}^{1121}+\sqrt{3}{\left|{1}_{\mathrm{L}}\rangle \right.}_{\mathrm{ABCD}}^{1122}\right)/2$ can be transformed to state ${\left|{\psi }_{4}\rangle \right.}_{\mathrm{ABCD}}$ by feed-forward (path switch), as shown in Fig. 5.

The encoding process shown in Fig. 5 comprises two parts (linear: arbitrary-BS, and nonlinear: final gate via XKNLs). To encode arbitrary quantum information for our purposes, communication, computation, teleportation, etc., onto the output state ${\left|{\psi }_{4}\rangle \right.}_{\mathrm{ABCD}}$ of the fourth gate, we can control the transmission rate (${\kappa }_{1}$) and reflection rate (${\kappa }_{2}$) of an arbitrary-BS, in which the operations are expressed as described in Fig. 5. Therefore, after applying the arbitrary-BS to state ${\left|{\psi }_{4}\rangle \right.}_{\mathrm{ABCD}}=\left({\left|{0}_{\mathrm{L}}\rangle \right.}_{\mathrm{ABCD}}^{1112}+\sqrt{3}{\left|{1}_{\mathrm{L}}\rangle \right.}_{\mathrm{ABCD}}^{1111}\right)/2$, the encoded (superposition of) state ${\left|{\psi }_{5}\rangle \right.}_{\mathrm{ABCD}}$ is expressed as

10 $$\begin{aligned}{\left|{\psi }_{5}\rangle \right.}_{\mathrm{ABCD}} & =\frac{1}{\sqrt{2}}\left[\frac{1}{\sqrt{3{\left|{\kappa }_{2}\right|}^{2}+{\left|{\kappa }_{1}\right|}^{2}}}\left({{\kappa }_{1}\left|{0}_{\mathrm{L}}\rangle \right.}_{\mathrm{ABCD}}^{1111}+\sqrt{3}{{\kappa }_{2}\left|{1}_{\mathrm{L}}\rangle \right.}_{\mathrm{ABCD}}^{1111}\right)-\frac{1}{\sqrt{3{\left|{\kappa }_{1}\right|}^{2}+{\left|{\kappa }_{2}\right|}^{2}}}\left({{\kappa }_{2}\left|{0}_{\mathrm{L}}\rangle \right.}_{\mathrm{ABCD}}^{1112}-\sqrt{3}{{\kappa }_{1}\left|{1}_{\mathrm{L}}\rangle \right.}_{\mathrm{ABCD}}^{1112}\right)\right] \\ & \equiv \frac{1}{\sqrt{2}}\left[\left({{\alpha }_{1}\left|{0}_{\mathrm{L}}\rangle \right.}_{\mathrm{ABCD}}^{1111}+{{\beta }_{1}\left|{1}_{\mathrm{L}}\rangle \right.}_{\mathrm{ABCD}}^{1111}\right)-\left({{\alpha }_{2}\left|{0}_{\mathrm{L}}\rangle \right.}_{\mathrm{ABCD}}^{1112}-{{\beta }_{2}\left|{1}_{\mathrm{L}}\rangle \right.}_{\mathrm{ABCD}}^{1112}\right)\right], \end{aligned}$$

where ${\left|{\alpha }_{i}\right|}^{2}+{\left|{\beta }_{i}\right|}^{2}=1$. ${\alpha }_{i}$ and ${\beta }_{i}$ denote the arbitrary information encoded by the arbitrary-BS. Subsequently, through the final gate, the pre-measurement (before PNR measurement) state ${\left|\psi_{5}^{^{\prime}}\rangle \right.}_{\mathrm{ABCD}}$ is expressed as

11 $$\begin{aligned}{\left|\psi_{5}^{^{\prime}}\rangle \right.}_{\mathrm{ABCD}} & =\frac{1}{\sqrt{2}}\left({{\alpha }_{1}\left|{0}_{\mathrm{L}}\rangle \right.}_{\mathrm{ABCD}}^{1111}+{{\beta }_{1}\left|{1}_{\mathrm{L}}\rangle \right.}_{\mathrm{ABCD}}^{1111}\right) \otimes {{\left|\alpha \rangle \right.}_{\mathrm{P}}^{\mathrm{a}}\left|0\rangle \right.}_{\mathrm{P}}^{\mathrm{b}} \\ & \quad -\frac{1}{\sqrt{2}}{e}^{-\frac{{\left(\alpha \mathrm{sin}\theta \right)}^{2}}{2}}\sum_{n=0}^{\infty }\frac{{\left(-i\alpha \mathrm{sin}\theta \right)}^{n}}{\sqrt{n!}}\left({{\alpha }_{2}\left|{0}_{\mathrm{L}}\rangle \right.}_{\mathrm{ABCD}}^{1112}-{{\beta }_{2}\left|{1}_{\mathrm{L}}\rangle \right.}_{\mathrm{ABCD}}^{1112}\right) \otimes {\left|\alpha \mathrm{cos}\theta \rangle \right.}_{\mathrm{P}}^{\mathrm{a}}{\left|n\rangle \right.}_{\mathrm{P}}^{\mathrm{b}}. \end{aligned}$$

After PNR measurement on path b, we can obtain the final state (single logical qubit information), which is the encoded arbitrary information, for the outcomes ($n=0$ or $n\ne 0$) of the PNR measurement, as follows:

12 $$\begin{aligned} & \left(n=0\right) \to {\left|{\psi }_{\mathrm{f}\_0}\rangle \right.}_{\mathrm{ABCD}}={{\alpha }_{1}\left|{0}_{\mathrm{L}}\rangle \right.}_{\mathrm{ABCD}}^{1111}+{{\beta }_{1}\left|{1}_{\mathrm{L}}\rangle \right.}_{\mathrm{ABCD}}^{1111}, \\ &\left(n\ne 0\right) \to {\left|{\psi }_{\mathrm{f}\_{\mathrm{n}}}\rangle \right.}_{\mathrm{ABCD}}={{\alpha }_{2}\left|{0}_{\mathrm{L}}\rangle \right.}_{\mathrm{ABCD}}^{1112}-{{\beta }_{2}\left|{1}_{\mathrm{L}}\rangle \right.}_{\mathrm{ABCD}}^{1112}, \end{aligned}$$

where the final state ${\left|{\psi }_{\mathrm{f}\_{\mathrm{n}}}\rangle \right.}_{\mathrm{ABCD}}$ ($n\ne 0$) can be converted to state ${\left|{\psi }_{\mathrm{f}\_0}\rangle \right.}_{\mathrm{ABCD}}$ ($n=0$) by applying unitary operations since the transmission rate, ${\kappa }_{1}$, and reflection rate, ${\kappa }_{2}$, of an arbitrary-BS are known. Consequently, for the single logical qubit information, the proposed procedure shown in Fig. 2 can encode the arbitrary quantum information (${\alpha }_{i}$ and ${\beta }_{i}$) onto four-photon decoherence-free states which are superposed state of $\left|{0}_{\mathrm{L}}\rangle \right.$ and $\left|{1}_{\mathrm{L}}\rangle \right.$, as shown in Eq. (12).

Herein, we propose a procedure comprising nonlinear optical gates, first, second, third, fourth, and final gates via XKNLs, and linear optical devices, including the arbitrary-BS, to encode single logical qubit information onto logical qubits (four-photon decoherence-free states) to protect quantum information against collective decoherence^32–34^. However, the nonlinear optical gates, which are components critical to this procedure (Fig. 2), cannot avoid the influences of photon loss and dephasing induced by the decoherence effect^57–59,63,65,72,73^. Therefore, we should derive the experimental condition to reduce the decoherence effect^57–59,63,65^ based on the master equation^74^ to quantify the efficiency and performance of the nonlinear optical gates.

## Quantification of efficiency and performance of nonlinear optical gates via XKNLs under decoherence effect

In the proposed procedure, the nonlinear optical gates, first, second, third, fourth, and final gates, using XKNLs are the most important components for generating decoherence-free states (logical qubits) and encoding arbitrary quantum information. Hence, these gates must be highly efficient and reliable when the proposed procedure is implemented in optical fibers^72,73^. Regarding the interaction of XKNLs of these gates, we should derive an experimental condition to decrease the decoherence effect^57–59,63,65^ using the master equation^74^, which can indicate the open quantum system, as follows:

13 $$\frac{\partial \rho \left(t\right)}{\partial t}=\frac{-i}{\hslash }\left[{H}_{K}, \rho \right]+\gamma \left[a\rho {a}^{+}-\frac{1}{2}\left\{{a}^{+}a\rho +{\rho a}^{+}a\right\}\right],$$

where ${H}_{K}=\hslash \chi {N}_{1}{N}_{2}$. The Lindblad operators are $\widehat{J}\rho =\gamma a\rho {a}^{+}$ and $\widehat{L}\rho =-\frac{\gamma }{2}\left({a}^{+}a\rho +{\rho a}^{+}a\right)$, where $\gamma$ is the energy decay rate. The solution to this equation is $\rho\left(t\right)=\mathrm{exp}\left[\left(\widehat{J}+\widehat{L}\right)t\right]\rho \left(0\right)$ for time $t \left(=\theta /\chi \right)$. Using this solution, we can exploit the process model, which can be used to formulate the influences of photon loss and dephasing of coherent parameters caused by the interaction (conditional phase shift) of XKNLs in nonlinear optical gates under the decoherence effect, as follows:

14 $${\left({\hat{D}}_{\Delta t}{\hat{X}}_{\Delta t}\right)}^{N}\left|1\rangle \right.\langle \left.0\right| \otimes \left|\alpha \rangle \right.\langle \left.\alpha \right|=\mathrm{exp}\left[-{\alpha }^{2}\left(1-{e}^{-\gamma\Delta t}\right)\sum_{n=1}^{N}\left(1-{e}^{in\Delta \theta }\right){e}^{-\gamma\Delta t\left(n-1\right)}\right]\left|1\rangle \right.\langle \left.0\right| \otimes \left|{\Lambda }_{t}\alpha {e}^{i\theta }\rangle \langle \left.{\Lambda }_{t}\alpha \right|\right.,$$

where ${{\hat{D}}_{t}{\hat{X}}_{t}=\left({\hat{D}}_{\Delta t}{\hat{X}}_{\Delta t}\right)}^{N}$ for $\theta =\chi t=\chi N\Delta t=N\Delta \theta$ owing to an arbitrarily small time $\Delta t \left(=t/N\right)$ for obtaining a good approximation^57–59,63,65^. Here, the decoherence ${\hat{D}}_{t}$ (photon loss and dephasing) and the rate, ${\Lambda }_{t}={e}^{-\gamma t/2}$ of the remaining photons in the probe beam can be calculated from the solution of the master equation shown in Eq. (13). Furthermore, the operation of the operator ${\hat{X}}_{t}$ (of the interaction of XKNLs) is expressed as ${\left({\hat{X}}_{\Delta t}\right)}^{N}\left|1\rangle \right.\langle \left.0\right| \otimes \left|\alpha \rangle \right.\langle \left.\alpha \right|=\left|1\rangle \right.\langle \left.0\right| \otimes \left|\alpha {e}^{iN\Delta \theta }\rangle \langle \left.\alpha \right|\right.=\left|1\rangle \right.\langle \left.0\right| \otimes \left|\alpha {e}^{i\theta }\rangle \langle \left.\alpha \right|\right.$ for $\left|1\rangle \right.$ (one photon) and $\left|0\rangle \right.$ (zero photon). Using this process model (decoherence: ${\hat{D}}_{t}$ and interaction of XKNL: ${\hat{X}}_{t}$) of Eqs. (13) and (14), we can quantify the influences of photon loss (${\Lambda }_{t}={e}^{-\gamma t/2}$) and dephasing of the value of the coherent parameter, i.e., the coefficient of the right-hand side of Eq. (14), induced by the decoherence effect. For the large phase shift ($\approx \pi$) at room temperature, researchers in Ref.^75^ demonstrated that the implementation of large phase shifts on a single-photon level probe pulse ($1.5\mathrm{ \mu s}$) is mediated by ${\mathrm{Rb}}^{87}$ vapor in a double-$\Lambda$atomic configuration to apply to quantum non-demolition detections ^10,12,14,16,18,56–64^. Also, for the practical realization of nonlinear optical gates, we should consider the experimental parameters and the features in the optical fibers^72,73^. In commercial fibers, which are pure silica-core fibers with a signal loss of 0.15 dB/km ($\chi /\gamma =0.0303$)^73^, a length of approximately 3000 km is required to acquire the magnitude of the conditional phase shift, $\theta =\pi$ from XKNLs. Hence, using the process model (Eqs. 13 and 14) with the experimental parameters and features of the optical fiber (length of 3000 km for $\theta =\pi$ and signal loss of 0.15 dB/km), we can analyze and quantify the efficiencies and performances of the nonlinear optical gates in the proposed procedure for encoding single logical qubit information (Fig. 2).

For the quantification of efficiency in ideal cases without the decoherence effect, we can obtain the error probabilities of nonlinear optical gates from the probabilities of measuring state ${\left|0\rangle \right.}_{\mathrm{P}}^{\mathrm{b}}$ (zero photon) in state ${\left|\pm i\alpha \mathrm{sin}\theta \rangle \right.}_{\mathrm{P}}^{\mathrm{b}}$ (Eqs. 4, 5, 6, 9, and 11), as follows: ${\mathrm{P}}_{\mathrm{err}}=\left[\mathrm{exp}\left(-{\alpha }^{2}{\mathrm{sin}}^{2}\theta \right)\right]/2\approx \left[\mathrm{exp}\left(-{\alpha }^{2}{\theta }^{2}\right)\right]/2$ for ${\alpha }^{2}{\mathrm{sin}}^{2}\theta \approx {\alpha }^{2}{\theta }^{2}$ with a strong amplitude of coherent state and small phase shift magnitude by the XKNL ($\alpha \gg 1$ and $\theta \ll 1$). If we do not consider the decoherence effect (ideal case), then the error probabilities of all nonlinear optical gates (first, second, third, fourth, and final) will be identical, as ${\mathrm{P}}_{\mathrm{err}}\approx \left[\mathrm{exp}\left(-{\alpha }^{2}{\theta }^{2}\right)\right]/2$. In addition, when the parameter is fixed as $\alpha \theta =2.5$, we can acquire highly efficient nonlinear optical gates (first, second, third, fourth, and final) because ${\mathrm{P}}_{\mathrm{err}}<{10}^{-3}$.

**Figure 6:**
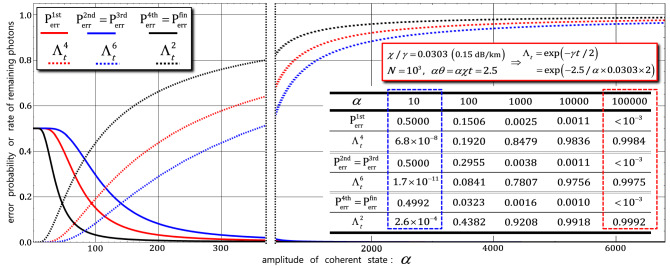
Error probabilities and rates of remaining photons in probe beam in practical case (under decoherence effect): Graph shows error probabilities (${\mathrm{P}}_{\mathrm{err}}^{1\mathrm{st}}$, ${\mathrm{P}}_{\mathrm{err}}^{2\mathrm{nd}}$, ${\mathrm{P}}_{\mathrm{err}}^{3\mathrm{rd}}$, ${\mathrm{P}}_{\mathrm{err}}^{4\mathrm{th}}$, and ${\mathrm{P}}_{\mathrm{err}}^{\mathrm{fin}}$) and rates (${\Lambda }_{t}={e}^{-\gamma t/2}$) of remaining photons of nonlinear optical gates (first, second, third, fourth, and final) for differences in amplitude ($\alpha$) of coherent state with fixed $\alpha \theta =2.5$ and signal loss of 0.15 dB/km ($\chi /\gamma =0.0303$). Values of error probabilities and rates of remaining photons in each gate are listed in table.

However, in the practical cases where the decoherence effect is considered, we should recalculate the error probabilities (${\mathrm{P}}_{\mathrm{err}}^{1\mathrm{st}}$, ${\mathrm{P}}_{\mathrm{err}}^{2\mathrm{nd}}$, ${\mathrm{P}}_{\mathrm{err}}^{3\mathrm{rd}}$, ${\mathrm{P}}_{\mathrm{err}}^{4\mathrm{th}}$, and ${\mathrm{P}}_{\mathrm{err}}^{\mathrm{fin}}$) including the photon loss (the rate ${\Lambda }_{t}={e}^{-\gamma t/2}$ of remaining photons) due to the decoherence effect, as follows:

15 $$\begin{aligned} & {\mathrm{P}}_{\mathrm{err}}^{1\mathrm{st}}=\left[\mathrm{exp}\left\{-{\Lambda }_{t}^{4}{\alpha }^{2}{\theta }^{2}\right\}\right]/2=\left[\mathrm{exp}\left\{-{e}^{-2\gamma t}\times {2.5}^{2}\right\}\right]/2=\left[\mathrm{exp}\left\{-{e}^{-2\left(\frac{2.5}{\alpha \times 0.0303}\right)}\times {2.5}^{2}\right\}\right]/2, \\ & {\mathrm{P}}_{\mathrm{err}}^{2\mathrm{nd}}={\mathrm{P}}_{\mathrm{err}}^{3\mathrm{rd}}=\left[\mathrm{exp}\left\{-{\Lambda }_{t}^{6}{\alpha }^{2}{\theta }^{2}\right\}\right]/2=\left[\mathrm{exp}\left\{-{e}^{-3\gamma t}\times {2.5}^{2}\right\}\right]/2=\left[\mathrm{exp}\left\{-{e}^{-3\left(\frac{2.5}{\alpha \times 0.0303}\right)}\times {2.5}^{2}\right\}\right]/2, \\ & {\mathrm{P}}_{\mathrm{err}}^{4\mathrm{th}}={\mathrm{P}}_{\mathrm{err}}^{\mathrm{fin}}=\left[\mathrm{exp}\left\{-{\Lambda }_{t}^{2}{\alpha }^{2}{\theta }^{2}\right\}\right]/2=\left[\mathrm{exp}\left\{-{e}^{-\gamma t}\times {2.5}^{2}\right\}\right]/2=\left[\mathrm{exp}\left\{-{e}^{-\left(\frac{2.5}{\alpha \times 0.0303}\right)}\times {2.5}^{2}\right\}\right]/2, \end{aligned}$$

where $\gamma t=2.5/(\alpha \times 0.0303)$ for ${\Lambda }_{t}={e}^{-\gamma t/2}$ with a fixed $\alpha \theta =\alpha \chi t=2.5$, and a signal loss of 0.15 dB/km ($\chi /\gamma =0.0303$) in optical fibers^73^. From these calculations, we can obtain the efficiency values of the nonlinear optical gates under the decoherence effect. Figure 6 shows the tendencies of the error probabilities (${\mathrm{P}}_{\mathrm{err}}^{1\mathrm{st}}$, ${\mathrm{P}}_{\mathrm{err}}^{2\mathrm{nd}}$, ${\mathrm{P}}_{\mathrm{err}}^{3\mathrm{rd}}$, ${\mathrm{P}}_{\mathrm{err}}^{4\mathrm{th}}$, and ${\mathrm{P}}_{\mathrm{err}}^{\mathrm{fin}}$) and rates (${\Lambda }_{t}^{4}$, ${\Lambda }_{t}^{6}$, and ${\Lambda }_{t}^{2}$) of the remaining photons of the gates (first, second, third, fourth, and final) in terms of the differences in the amplitude of the coherent state ($\alpha$) with the following parameters: signal loss of 0.15 dB/km ($\chi /\gamma =0.0303$), $\alpha \theta =\alpha \chi t=2.5$, and $N={10}^{3}$. In addition, the values of error probabilities (${\mathrm{P}}_{\mathrm{err}}^{1\mathrm{st}}$, ${\mathrm{P}}_{\mathrm{err}}^{2\mathrm{nd}}$, ${\mathrm{P}}_{\mathrm{err}}^{3\mathrm{rd}}$, ${\mathrm{P}}_{\mathrm{err}}^{4\mathrm{th}}$, and ${\mathrm{P}}_{\mathrm{err}}^{\mathrm{fin}}$) and rates (${\Lambda }_{t}^{4}$, ${\Lambda }_{t}^{6}$, and ${\Lambda }_{t}^{2}$) of the remaining photons of the gates were calculated for the amplitudes, $\alpha$=$10$, $100$, $1000$, $10000$, and $100000$, of the coherent state, as listed on Table (in Fig. 6). Compared with the dotted-blue box and dotted-red box in Table, we can conclude that high efficiencies, i.e., ${\mathrm{P}}_{\mathrm{err}}<{10}^{-3}$ (and low rate, ${\Lambda }_{t}\approx 1.0$ of photon loss), can be achieved in the nonlinear optical gates by employing a strong amplitude of coherent state ($\alpha \gg 10$) under the decoherence effect.

Moreover, we should analyze the performances, the influence of dephasing, of the nonlinear optical gates, in addition to the efficiencies which are quantified by error probabilities by photon loss in Fig. 6. To quantify the influences of dephasing of coherent parameters induced by the decoherence effect, we require a process model (Eqs. 13 and 14) that can describe the dynamics of the interactions of XKNLs (${\hat{X}}_{t}$) and the decoherence effect (${\hat{D}}_{t}$) to analyze the output states from the nonlinear optical gates.

**Figure 7:**
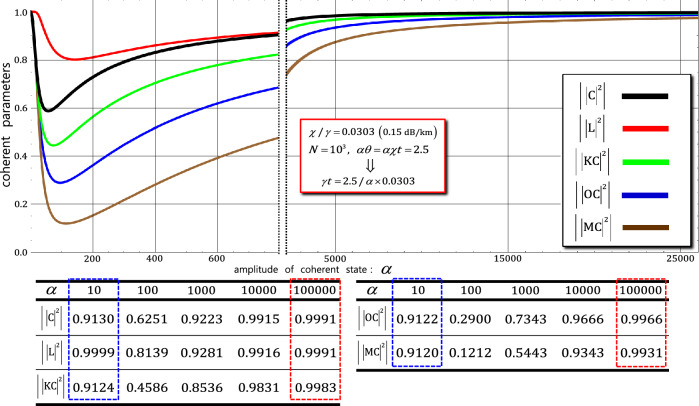
Trends and values of coherent parameters in output states of first, fourth, and final gates by dephasing (decoherence effect): Graph represents coherent parameters in output states of the nonlinear optical gates (first, fourth, and final) for differences in amplitude ($\alpha$) of coherent state with signal loss of 0.15 dB/km ($\chi /\gamma =0.0303$), $\alpha \theta =2.5$ (${\mathrm{P}}_{\mathrm{err}}<{10}^{-3}$), and $N={10}^{3}$. Values of coherent parameters in output states are listed in table.

In the first, fourth, and final gates, using the process model’s formula (Eqs. 13 and 14), the output states ${\left|\psi_{0}^{^{\prime}}\rangle \right.}_{\mathrm{ABCD}}$ in Eq. (4), ${\left|\psi_{3}^{{^{\prime\prime}}}\rangle \right.}_{\mathrm{ABCD}}$ in Eq. (9), and ${\left|\psi_{5}^{^{\prime}}\rangle \right.}_{\mathrm{ABCD}}$ in Eq. (11) can be expressed as density matrices ${\rho }_{0}^{^{\prime}}$ of the first gate, ${\rho }_{3}^{{^{\prime\prime}}}$ of the fourth gate, and ${\rho }_{5}^{^{\prime}}$ of the final gate, respectively, to determine the dephasing of coherent parameters, as follows:

16 $${\rho }_{0}^{^{\prime}}=\frac{1}{4}\left(\begin{array}{cccc}1& {\left|\mathrm{CK}\right|}^{2}& {\left|\mathrm{L}\right|}^{2}& {\left|\mathrm{CO}\right|}^{2}\\ {\left|\mathrm{CK}\right|}^{2}& 1& {\left|\mathrm{CO}\right|}^{2}& {\left|\mathrm{L}\right|}^{2}\\ {\left|\mathrm{L}\right|}^{2}& {\left|\mathrm{CO}\right|}^{2}& 1& {\left|\mathrm{CM}\right|}^{2}\\ {\left|\mathrm{CO}\right|}^{2}& {\left|\mathrm{L}\right|}^{2}& {\left|\mathrm{CM}\right|}^{2}& 1\end{array}\right),\;\; {\rho }_{3}^{{^{\prime\prime}}}={\rho }_{5}^{^{\prime}}=\frac{1}{2}\left(\begin{array}{cc}1& {\left|\mathrm{C}\right|}^{2}\\ {\left|\mathrm{C}\right|}^{2}& 1\end{array}\right),$$

where the bases of ${\rho }_{0}^{{{\prime}}}$ are the states in ${\left|L\rangle \right.}_{\mathrm{A}}^{1}{\left|L\rangle \right.}_{\mathrm{B}}^{1}{\left|R\rangle \right.}_{\mathrm{C}}^{1}{\left|R\rangle \right.}_{\mathrm{D}}^{1}{\left|{\Lambda }_{t}^{2}\alpha \rangle \right.}_{\mathrm{P}}^{\mathrm{a}}{\left|0\rangle \right.}_{\mathrm{P}}^{\mathrm{b}}$, ${\left|L\rangle \right.}_{\mathrm{A}}^{1}{\left|L\rangle \right.}_{\mathrm{B}}^{1}{\left|R\rangle \right.}_{\mathrm{C}}^{2}{\left|R\rangle \right.}_{\mathrm{D}}^{2}{\left|{\Lambda }_{t}^{2}\alpha \rangle \right.}_{\mathrm{P}}^{\mathrm{a}}{\left|0\rangle \right.}_{\mathrm{P}}^{\mathrm{b}}$, ${\left|L\rangle \right.}_{\mathrm{A}}^{1}{\left|L\rangle \right.}_{\mathrm{B}}^{1}{\left|R\rangle \right.}_{\mathrm{C}}^{1}{\left|R\rangle \right.}_{\mathrm{D}}^{2}{{\left|{\Lambda }_{t}^{2}\alpha \mathrm{cos}\theta \rangle \right.}_{\mathrm{P}}^{\mathrm{a}}\left|{i\Lambda }_{t}^{2}\alpha \mathrm{sin}\theta \rangle \right.}_{\mathrm{P}}^{\mathrm{b}}$, and ${\left|L\rangle \right.}_{\mathrm{A}}^{1}{\left|L\rangle \right.}_{\mathrm{B}}^{1}{\left|R\rangle \right.}_{\mathrm{C}}^{1}{\left|R\rangle \right.}_{\mathrm{D}}^{2}{{\left|{\Lambda }_{t}^{2}\alpha \mathrm{cos}\theta \rangle \right.}_{\mathrm{P}}^{\mathrm{a}}\left|-{i\Lambda }_{t}^{2}\alpha \mathrm{sin}\theta \rangle \right.}_{\mathrm{P}}^{\mathrm{b}}$; the bases of ${\rho }_{3}^{\mathrm{^{\prime}}\mathrm{^{\prime}}}$ are the states in $\left(\frac{1}{2}{\left|{0}_{\mathrm{L}}\rangle \right.}_{\mathrm{ABCD}}^{1112}+\frac{\sqrt{3}}{2}{\left|{1}_{\mathrm{L}}\rangle \right.}_{\mathrm{ABCD}}^{1111}\right){{\left|{\Lambda }_{t}\alpha \rangle \right.}_{\mathrm{P}}^{\mathrm{a}}\left|0\rangle \right.}_{\mathrm{P}}^{\mathrm{b}}$ and $\left(\frac{1}{2}{\left|{0}_{\mathrm{L}}\rangle \right.}_{\mathrm{ABCD}}^{1121}+\frac{\sqrt{3}}{2}{\left|{1}_{\mathrm{L}}\rangle \right.}_{\mathrm{ABCD}}^{1122}\right){{\left|{\Lambda }_{t}\alpha \mathrm{cos}\theta \rangle \right.}_{\mathrm{P}}^{\mathrm{a}}\left|-i{\Lambda }_{t}\alpha \mathrm{sin}\theta \rangle \right.}_{\mathrm{P}}^{\mathrm{b}}$; the bases of ${\rho }_{5}^{\mathrm{^{\prime}}}$ are the states in $\left({{\alpha }_{1}\left|{0}_{\mathrm{L}}\rangle \right.}_{\mathrm{ABCD}}^{1111}+{{\beta }_{1}\left|{1}_{\mathrm{L}}\rangle \right.}_{\mathrm{ABCD}}^{1111}\right){{\left|{\Lambda }_{t}\alpha \rangle \right.}_{\mathrm{P}}^{\mathrm{a}}\left|0\rangle \right.}_{\mathrm{P}}^{\mathrm{b}}$ and $\left({{\alpha }_{2}\left|{0}_{\mathrm{L}}\rangle \right.}_{\mathrm{ABCD}}^{1112}-{{\beta }_{2}\left|{1}_{\mathrm{L}}\rangle \right.}_{\mathrm{ABCD}}^{1112}\right){{\left|{\Lambda }_{t}\alpha \mathrm{cos}\theta \rangle \right.}_{\mathrm{P}}^{\mathrm{a}}\left|-i{\Lambda }_{t}\alpha \mathrm{sin}\theta \rangle \right.}_{\mathrm{P}}^{\mathrm{b}}$ from left to right and top to bottom. Based on the process model expressed in Eq. (14), the coherent parameters in the density matrices (${\rho }_{0}^{\mathrm{^{\prime}}}$, ${\rho }_{3}^{\mathrm{^{\prime}}\mathrm{^{\prime}}}$, and ${\rho }_{5}^{\mathrm{^{\prime}}}$) are expressed as

$$\mathrm{C}=\mathrm{exp}\left[-\left({\alpha }/\sqrt{2}\right)^{2}\left(1-{e}^{-\gamma\Delta t}\right){\sum }_{n=1}^{N}\left(1-{e}^{in\Delta \theta }\right){e}^{-\gamma\Delta t\left(n-1\right)}\right],$$

$$\mathrm{L}=\mathrm{exp}\left[-\left({\alpha }/\sqrt{2}\right)^{2}\left({e}^{-\gamma t}\right)\left(1-{e}^{-\gamma\Delta t}\right){\sum }_{n=1}^{N}\left(1-{e}^{in\Delta \theta }\right){e}^{-\gamma\Delta t\left(n-1\right)}\right],$$

$$\mathrm{K}=\mathrm{exp}\left[-\left({\alpha }/\sqrt{2}\right)^{2}\left({e}^{-\gamma t}\right)\left(1-{e}^{-\gamma\Delta t}\right){\sum }_{n=1}^{N}{\left(1-{{e}^{i\theta }\cdot e}^{-in\Delta \theta }\right)e}^{-\gamma\Delta t\left(n-1\right)}\right],$$

$$\mathrm{M}=\mathrm{exp}\left[-\left({\alpha }/\sqrt{2}\right)^{2}\left({e}^{-\gamma t}\right)\left(1-{e}^{-\gamma\Delta t}\right){\sum }_{n=1}^{N}{\left(1-{{e}^{i\theta }\cdot e}^{in\Delta \theta }\right)e}^{-\gamma\Delta t\left(n-1\right)}\right],$$

17 $$\mathrm{O}=\mathrm{exp}\left[-\left({\alpha }/\sqrt{2}\right)^{2}\left({e}^{-\gamma t}\right)\left(1-{e}^{-\gamma\Delta t}\right)\left(1-{e}^{i\theta }\right){\sum }_{n=1}^{N}{e}^{-\gamma\Delta t\left(n-1\right)}\right],$$

where for $\theta =\chi t=\chi N\Delta t=N\Delta \theta$ and $\alpha \in {\mathbb{R}}$ with an arbitrarily small time $\Delta t=t/N$ (for a good approximation^57–59,63,65^). In the density matrices (${\rho }_{0}^{^{\prime}}$, ${\rho }_{3}^{{^{\prime\prime}}}$, and ${\rho }_{5}^{^{\prime}}$), the off-diagonal terms refer to the coherent parameters ($\mathrm{C}$, $\mathrm{L}$, $\mathrm{K}$, $\mathrm{M}$, and $\mathrm{O}$) that can be used to evaluate the degrees of a mixed state and quantify the influences of dephasing. For example, if the values of the coherent parameters (off-diagonal terms) decrease by dephasing (decoherence effect), then the output states (${\rho }_{0}^{^{\prime}}$ of the first gate, ${\rho }_{3}^{{^{\prime\prime}}}$ of the fourth gate, and ${\rho }_{5}^{^{\prime}}$ of the final gate) evolve into mixed states, the ensemble of classical states. Therefore, to obtain reliable performances from the nonlinear optical gates, first, fourth, and final gates, the values of the coherent parameters should be retrained to approach 1 for the pure quantum state against dephasing by the decoherence effect. Figure 7 shows the tendencies of the coherent parameters ($\left|{\left|\mathrm{C}\right|}^{2}\right|$, $\left|{\left|\mathrm{L}\right|}^{2}\right|$, $\left|{\left|\mathrm{KC}\right|}^{2}\right|$, $\left|{\left|\mathrm{OC}\right|}^{2}\right|$, and $\left|{\left|\mathrm{MC}\right|}^{2}\right|$) in the density matrices (${\rho }_{0}^{^{\prime}}$, ${\rho }_{3}^{{^{\prime\prime}}}$, and ${\rho }_{5}^{^{\prime}}$) of the first, fourth, and final gates for the amplitude of the coherent state, probe beam: $\alpha$, with the following parameters: signal loss of 0.15 dB/km ($\chi /\gamma =0.0303$), $\alpha \theta =\alpha \chi t=2.5$ (${\mathrm{P}}_{\mathrm{err}}<{10}^{-3}$), and $N={10}^{3}$ under the decoherence effect. In addition, based on the table shown in Fig. 7, we calculated the values of the coherent parameters based on the differences in the amplitudes ($\alpha$= $10$, $100$, $1000$, $10000$, and $100000$) of the probe beams. As shown in Fig. 7, if the amplitude of the coherent state (probe beam) increases ($\alpha \gg 100$), then all values of the coherent parameters are approximately 1. Hence, by employing the strong (large amplitude) coherent state, we can maintain the output state (${\rho }_{0}^{^{\prime}}$, ${\rho }_{3}^{{^{\prime\prime}}}$, and ${\rho }_{5}^{^{\prime}}$) of the first, fourth, and final gates to pure quantum states (prevention of off-diagonal terms in the density matrices, Eq. 16) against the influence of dephasing induced by the decoherence effect. Herein, as shown by the dotted blue box, the values of coherent parameters are high (> 0.9) when the amplitude of the coherent state is small ($\alpha =10$), compared with the values within the dotted-red box in Fig. 7. However, in the small amplitude range ($\alpha <10$) of the coherent state, we could not acquire a high rate of highly efficient remaining photons in the first, fourth, and final gates, as shown in Fig. 6 (dotted-blue box). Hence, for high efficiencies (low error probabilities) and reliable performances (preserved pure quantum states in the output) in the first, fourth, and final gates, we should utilize the strong coherent state (probe beam) to reduce the influences of photon loss and dephasing.

**Figure 8:**
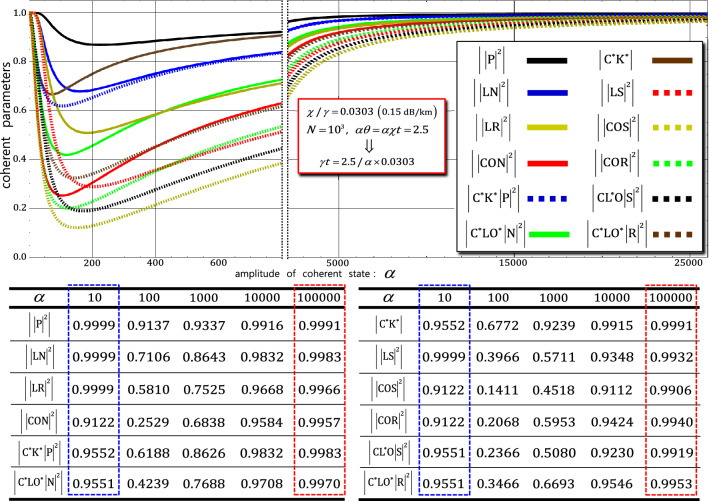
Trends and values of coherent parameters in output states of second and third gates by dephasing (decoherence effect): Graph represents coherent parameters in output states of nonlinear optical gates (second and third) for differences in amplitude ($\alpha$) of coherent state with signal loss of 0.15 dB/km ($\chi /\gamma =0.0303$), $\alpha \theta =2.5$ (${\mathrm{P}}_{\mathrm{err}}<{10}^{-3}$), and $N={10}^{3}$. Values of coherent parameters in output states are listed in table.

In the second and third gates, using the process model’s formula (Eqs. 13 and 14), the output states (${\left|\psi_{1}^{^{\prime}}\rangle \right.}_{\mathrm{ABCD}}$ in Eq. 5 and ${\left|\psi_{2}^{^{\prime}}\rangle \right.}_{\mathrm{ABCD}}$ in Eq. 6) can be expressed as density matrices ${\rho }_{1}^{^{\prime}}$ of the second gate and ${\rho }_{2}^{^{\prime}}$ of the third gate, as follows:

18 $${\rho }_{1}^{^{\prime}}={\rho }_{2}^{^{\prime}}=\frac{1}{8}\left(\begin{array}{cccccccc}1& {\left|\mathrm{LN}\right|}^{2}& {\mathrm{C}}^{*}{\mathrm{K}}^{*}& {\mathrm{C}}^{*}{\mathrm{LO}}^{*}{\left|\mathrm{N}\right|}^{2}& {\left|\mathrm{P}\right|}^{2}& {\mathrm{C}}^{*}{\mathrm{K}}^{*}{\left|\mathrm{P}\right|}^{2}& {\left|\mathrm{LR}\right|}^{2}& {\mathrm{C}}^{*}{\mathrm{LO}}^{*}{\left|\mathrm{R}\right|}^{2}\\ {\left|\mathrm{LN}\right|}^{2}& 1& {\mathrm{C}}^{*}{\mathrm{LO}}^{*}{\left|\mathrm{N}\right|}^{2}& {\mathrm{C}}^{*}{\mathrm{K}}^{*}& {\left|\mathrm{LR}\right|}^{2}& {\mathrm{C}}^{*}{\mathrm{LO}}^{*}{\left|\mathrm{R}\right|}^{2}& {\left|\mathrm{P}\right|}^{2}& {\mathrm{C}}^{*}{\mathrm{K}}^{*}{\left|\mathrm{P}\right|}^{2}\\ \mathrm{CK}& {\mathrm{CL}}^{*}\mathrm{O}{\left|\mathrm{N}\right|}^{2}& 1& {\left|\mathrm{CON}\right|}^{2}& \mathrm{CK}{\left|\mathrm{P}\right|}^{2}& {\left|\mathrm{P}\right|}^{2}& {\mathrm{CL}}^{*}\mathrm{O}{\left|\mathrm{R}\right|}^{2}& {\left|\mathrm{COR}\right|}^{2}\\ {\mathrm{CL}}^{*}\mathrm{O}{\left|\mathrm{N}\right|}^{2}& \mathrm{CK}& {\left|\mathrm{CON}\right|}^{2}& 1& {\mathrm{CL}}^{*}\mathrm{O}{\left|\mathrm{R}\right|}^{2}& {\left|\mathrm{COR}\right|}^{2}& \mathrm{CK}{\left|\mathrm{P}\right|}^{2}& {\left|\mathrm{P}\right|}^{2}\\ {\left|\mathrm{P}\right|}^{2}& {\left|\mathrm{LR}\right|}^{2}& {\mathrm{C}}^{*}{\mathrm{K}}^{*}{\left|\mathrm{P}\right|}^{2}& {\mathrm{C}}^{*}{\mathrm{LO}}^{*}{\left|\mathrm{R}\right|}^{2}& 1& \mathrm{CK}& {\left|\mathrm{LS}\right|}^{2}& {\mathrm{C}}^{*}{\mathrm{LO}}^{*}{\left|\mathrm{S}\right|}^{2}\\ \mathrm{CK}{\left|\mathrm{P}\right|}^{2}& {\mathrm{CL}}^{*}\mathrm{O}{\left|\mathrm{R}\right|}^{2}& {\left|\mathrm{P}\right|}^{2}& {\left|\mathrm{COR}\right|}^{2}& {\mathrm{C}}^{*}{\mathrm{K}}^{*}& 1& {\mathrm{CL}}^{*}\mathrm{O}{\left|\mathrm{S}\right|}^{2}& {\left|\mathrm{COS}\right|}^{2}\\ {\left|\mathrm{LR}\right|}^{2}& {\left|\mathrm{P}\right|}^{2}& {\mathrm{C}}^{*}{\mathrm{LO}}^{*}{\left|\mathrm{R}\right|}^{2}& {\mathrm{C}}^{*}{\mathrm{K}}^{*}{\left|\mathrm{P}\right|}^{2}& {\left|\mathrm{LS}\right|}^{2}& {\mathrm{C}}^{*}{\mathrm{LO}}^{*}{\left|\mathrm{S}\right|}^{2}& 1& {\mathrm{C}}^{*}{\mathrm{K}}^{*}\\ {\mathrm{CL}}^{*}\mathrm{O}{\left|\mathrm{R}\right|}^{2}& \mathrm{CK}{\left|\mathrm{P}\right|}^{2}& {\left|\mathrm{COR}\right|}^{2}& {\left|\mathrm{P}\right|}^{2}& {\mathrm{CL}}^{*}\mathrm{O}{\left|\mathrm{S}\right|}^{2}& {\left|\mathrm{COS}\right|}^{2}& \mathrm{CK}& 1\end{array}\right),$$

where the bases of ${\rho }_{1}^{\mathrm{^{\prime}}}$ are the states in ${\left|H\rangle \right.}_{\mathrm{A}}^{1}{\left|L\rangle \right.}_{\mathrm{B}}^{1}{\left|V\rangle \right.}_{\mathrm{C}}^{1}{\left|R\rangle \right.}_{\mathrm{D}}^{1}{\left|{\Lambda }_{t}^{3}\alpha \rangle \right.}_{\mathrm{P}}^{\mathrm{a}}{\left|0\rangle \right.}_{\mathrm{P}}^{\mathrm{b}}$, ${\left|H\rangle \right.}_{\mathrm{A}}^{1}{\left|L\rangle \right.}_{\mathrm{B}}^{1}{\left|R\rangle \right.}_{\mathrm{C}}^{2}{\left|V\rangle \right.}_{\mathrm{D}}^{2}{\left|{\Lambda }_{t}^{3}\alpha \rangle \right.}_{\mathrm{P}}^{\mathrm{a}}{\left|0\rangle \right.}_{\mathrm{P}}^{\mathrm{b}}$, ${\left|V\rangle \right.}_{\mathrm{A}}^{1}{\left|L\rangle \right.}_{\mathrm{B}}^{1}{\left|H\rangle \right.}_{\mathrm{C}}^{1}{\left|R\rangle \right.}_{\mathrm{D}}^{1}{\left|{\Lambda }_{t}^{3}\alpha \rangle \right.}_{\mathrm{P}}^{\mathrm{a}}{\left|0\rangle \right.}_{\mathrm{P}}^{\mathrm{b}}$, ${\left|V\rangle \right.}_{\mathrm{A}}^{1}{\left|L\rangle \right.}_{\mathrm{B}}^{1}{\left|R\rangle \right.}_{\mathrm{C}}^{1}{\left|H\rangle \right.}_{\mathrm{D}}^{1}{\left|{\Lambda }_{t}^{3}\alpha \rangle \right.}_{\mathrm{P}}^{\mathrm{a}}{\left|0\rangle \right.}_{\mathrm{P}}^{\mathrm{b}}$, ${\left|H\rangle \right.}_{\mathrm{A}}^{1}{\left|L\rangle \right.}_{\mathrm{B}}^{1}{\left|H\rangle \right.}_{\mathrm{C}}^{1}{\left|R\rangle \right.}_{\mathrm{D}}^{1}{\left|{\Lambda }_{t}^{3}\alpha \mathrm{cos}\theta \rangle \right.}_{\mathrm{P}}^{\mathrm{a}}{\left|{i\Lambda }_{t}^{3}\alpha \mathrm{sin}\theta \rangle \right.}_{\mathrm{P}}^{\mathrm{b}}$, ${\left|H\rangle \right.}_{\mathrm{A}}^{1}{\left|L\rangle \right.}_{\mathrm{B}}^{1}{\left|R\rangle \right.}_{\mathrm{C}}^{2}{\left|H\rangle \right.}_{\mathrm{D}}^{2}{\left|{\Lambda }_{t}^{3}\alpha \mathrm{cos}\theta \rangle \right.}_{\mathrm{P}}^{\mathrm{a}}{\left|{i\Lambda }_{t}^{3}\alpha \mathrm{sin}\theta \rangle \right.}_{\mathrm{P}}^{\mathrm{b}}$, ${\left|V\rangle \right.}_{\mathrm{A}}^{1}{\left|L\rangle \right.}_{\mathrm{B}}^{1}{\left|V\rangle \right.}_{\mathrm{C}}^{1}{\left|R\rangle \right.}_{\mathrm{D}}^{1}{\left|{\Lambda }_{t}^{3}\alpha \mathrm{cos}\theta \rangle \right.}_{\mathrm{P}}^{\mathrm{a}}{\left|-{i\Lambda }_{t}^{3}\alpha \mathrm{sin}\theta \rangle \right.}_{\mathrm{P}}^{\mathrm{b}}$, and ${\left|V\rangle \right.}_{\mathrm{A}}^{1}{\left|L\rangle \right.}_{\mathrm{B}}^{1}{\left|R\rangle \right.}_{\mathrm{C}}^{2}{\left|V\rangle \right.}_{\mathrm{D}}^{2}{\left|{\Lambda }_{t}^{3}\alpha \mathrm{cos}\theta \rangle \right.}_{\mathrm{P}}^{\mathrm{a}}{\left|{-i\Lambda }_{t}^{3}\alpha \mathrm{sin}\theta \rangle \right.}_{\mathrm{P}}^{\mathrm{b}}$; the bases of ${\rho }_{2}^{\mathrm{^{\prime}}}$ are the states in $\frac{1}{\sqrt{2}}{\left|H\rangle \right.}_{\mathrm{A}}^{1}{\left|H\rangle \right.}_{\mathrm{B}}^{1}\left({\left|V\rangle \right.}_{\mathrm{C}}^{1}{\left|V\rangle \right.}_{\mathrm{D}}^{1}+{\left|V\rangle \right.}_{\mathrm{C}}^{2}{\left|V\rangle \right.}_{\mathrm{D}}^{2}\right){\left|{\Lambda }_{t}^{3}\alpha \rangle \right.}_{\mathrm{P}}^{\mathrm{a}}{\left|0\rangle \right.}_{\mathrm{P}}^{\mathrm{b}}$, $\frac{1}{\sqrt{2}}{\left|V\rangle \right.}_{\mathrm{A}}^{1}{\left|H\rangle \right.}_{\mathrm{B}}^{1}\left({\left|H\rangle \right.}_{\mathrm{C}}^{1}{\left|V\rangle \right.}_{\mathrm{D}}^{1}+{\left|V\rangle \right.}_{\mathrm{C}}^{2}{\left|H\rangle \right.}_{\mathrm{D}}^{2}\right){\left|{\Lambda }_{t}^{3}\alpha \rangle \right.}_{\mathrm{P}}^{\mathrm{a}}{\left|0\rangle \right.}_{\mathrm{P}}^{\mathrm{b}}$, $\frac{1}{\sqrt{2}}{\left|V\rangle \right.}_{\mathrm{A}}^{1}{\left|V\rangle \right.}_{\mathrm{B}}^{1}\left({\left|H\rangle \right.}_{\mathrm{C}}^{1}{\left|H\rangle \right.}_{\mathrm{D}}^{1}+{\left|H\rangle \right.}_{\mathrm{C}}^{2}{\left|H\rangle \right.}_{\mathrm{D}}^{2}\right){\left|{\Lambda }_{t}^{3}\alpha \rangle \right.}_{\mathrm{P}}^{\mathrm{a}}{\left|0\rangle \right.}_{\mathrm{P}}^{\mathrm{b}}$, $\frac{1}{\sqrt{2}}{\left|H\rangle \right.}_{\mathrm{A}}^{1}{\left|V\rangle \right.}_{\mathrm{B}}^{1}\left({\left|H\rangle \right.}_{\mathrm{C}}^{2}{\left|V\rangle \right.}_{\mathrm{D}}^{2}+{\left|V\rangle \right.}_{\mathrm{C}}^{1}{\left|H\rangle \right.}_{\mathrm{D}}^{1}\right){\left|{\Lambda }_{t}^{3}\alpha \rangle \right.}_{\mathrm{P}}^{\mathrm{a}}{\left|0\rangle \right.}_{\mathrm{P}}^{\mathrm{b}}$, $\frac{1}{\sqrt{2}}{\left|H\rangle \right.}_{\mathrm{A}}^{1}{\left|H\rangle \right.}_{\mathrm{B}}^{1}\left({\left|V\rangle \right.}_{\mathrm{C}}^{1}{\left|H\rangle \right.}_{\mathrm{D}}^{1}+{\left|H\rangle \right.}_{\mathrm{C}}^{2}{\left|V\rangle \right.}_{\mathrm{D}}^{2}\right){\left|{\Lambda }_{t}^{3}\alpha \mathrm{cos}\theta \rangle \right.}_{\mathrm{P}}^{\mathrm{a}}{\left|{i\Lambda }_{t}^{3}\alpha \mathrm{sin}\theta \rangle \right.}_{\mathrm{P}}^{\mathrm{b}}$, $\frac{1}{\sqrt{2}}{\left|V\rangle \right.}_{\mathrm{A}}^{1}{\left|H\rangle \right.}_{\mathrm{B}}^{1}\left({\left|H\rangle \right.}_{\mathrm{C}}^{1}{\left|H\rangle \right.}_{\mathrm{D}}^{1}+{\left|H\rangle \right.}_{\mathrm{C}}^{2}{\left|H\rangle \right.}_{\mathrm{D}}^{2}\right){\left|{\Lambda }_{t}^{3}\alpha \mathrm{cos}\theta \rangle \right.}_{\mathrm{P}}^{\mathrm{a}}{\left|{i\Lambda }_{t}^{3}\alpha \mathrm{sin}\theta \rangle \right.}_{\mathrm{P}}^{\mathrm{b}}$, $\frac{1}{\sqrt{2}}{\left|V\rangle \right.}_{\mathrm{A}}^{1}{\left|V\rangle \right.}_{\mathrm{B}}^{1}\left({\left|H\rangle \right.}_{\mathrm{C}}^{1}{\left|V\rangle \right.}_{\mathrm{D}}^{1}+{\left|V\rangle \right.}_{\mathrm{C}}^{2}{\left|H\rangle \right.}_{\mathrm{D}}^{2}\right){\left|{\Lambda }_{t}^{3}\alpha \mathrm{cos}\theta \rangle \right.}_{\mathrm{P}}^{\mathrm{a}}{\left|{-i\Lambda }_{t}^{3}\alpha \mathrm{sin}\theta \rangle \right.}_{\mathrm{P}}^{\mathrm{b}}$, and $\frac{1}{\sqrt{2}}{\left|H\rangle \right.}_{\mathrm{A}}^{1}{\left|V\rangle \right.}_{\mathrm{B}}^{1}\left({\left|V\rangle \right.}_{\mathrm{C}}^{1}{\left|V\rangle \right.}_{\mathrm{D}}^{1}+{\left|V\rangle \right.}_{\mathrm{C}}^{2}{\left|V\rangle \right.}_{\mathrm{D}}^{2}\right){\left|{\Lambda }_{t}^{3}\alpha \mathrm{cos}\theta \rangle \right.}_{\mathrm{P}}^{\mathrm{a}}{\left|{-i\Lambda }_{t}^{3}\alpha \mathrm{sin}\theta \rangle \right.}_{\mathrm{P}}^{\mathrm{b}}$ from left to right and top to bottom. The coherent parameters ($\mathrm{C}$, $\mathrm{L}$, $\mathrm{K}$, $\mathrm{M}$, and $\mathrm{O}$) can be calculated using Eq. (17). The other coherent parameters ($\mathrm{P}$, $\mathrm{N}$, $\mathrm{S}$, and $\mathrm{R}$) in density matrices (${\rho }_{1}^{\mathrm{^{\prime}}}$ and ${\rho }_{2}^{\mathrm{^{\prime}}}$) can be calculated from the process model (Eq. 14), as follows:

$$\mathrm{P}=\mathrm{exp}\left[-\left({\alpha }/\sqrt{2}\right)^{2}\left({e}^{-2\gamma t}\right)\left(1-{e}^{-\gamma\Delta t}\right){\sum }_{n=1}^{N}\left(1-{e}^{in\Delta \theta }\right){e}^{-\gamma\Delta t\left(n-1\right)}\right],$$

$$\mathrm{N}=\mathrm{exp}\left[-\left({\alpha }/\sqrt{2}\right)^{2}\left({e}^{-2\gamma t}\right)\left(1-{e}^{-\gamma\Delta t}\right){\sum }_{n=1}^{N}{\left(1-{{e}^{i\theta }\cdot e}^{-in\Delta \theta }\right)e}^{-\gamma\Delta t\left(n-1\right)}\right],$$

$$\mathrm{S}=\mathrm{exp}\left[-\left({\alpha }/\sqrt{2}\right)^{2}\left({e}^{-2\gamma t}\right)\left(1-{e}^{-\gamma\Delta t}\right){\sum }_{n=1}^{N}{\left(1-{{e}^{i\theta }\cdot e}^{in\Delta \theta }\right)e}^{-\gamma\Delta t\left(n-1\right)}\right],$$

19 $$\mathrm{R}=\mathrm{exp}\left[-\left({\alpha }/\sqrt{2}\right)^{2}\left({e}^{-2\gamma t}\right)\left(1-{e}^{-\gamma\Delta t}\right)\left(1-{e}^{i\theta }\right){\sum }_{n=1}^{N}{e}^{-\gamma\Delta t\left(n-1\right)}\right],$$

where $\theta =\chi t=\chi N\Delta t=N\Delta \theta$ for an arbitrarily small time $\Delta t \left(=t/N\right)$ with $\alpha \in {\mathbb{R}}$. For the experimental condition to preserve the values of coherent parameters to 1 (pure quantum states) under the decoherence effect, we can determine the tendencies of the coherent parameters, off-diagonal terms, in the density matrices (${\rho }_{1}^{^{\prime}}$ and ${\rho }_{2}^{^{\prime}}$) of the second and third gates for the amplitude of the coherent state (probe beam: $\alpha$) using the following parameters: signal loss of 0.15 dB/km ($\chi /\gamma =0.0303$), $\alpha \theta =\alpha \chi t=2.5$ (${\mathrm{P}}_{\mathrm{err}}<{10}^{-3}$), and $N={10}^{3}$, as shown in Fig. 8. In addition, the values of the coherent parameters are listed on Table (in Fig. 8) based on the differences in the amplitudes, $\alpha$ = $10$, $100$, $1000$, $10000$, and $100000$, of the probe beams. When the amplitude of the coherent state (probe beam) increased ($\alpha \gg 100$), all values of the coherent parameters were approximately 1, similar to the coherent parameters in ${\rho }_{0}^{^{\prime}}$, ${\rho }_{3}^{{^{\prime\prime}}}$, and ${\rho }_{5}^{^{\prime}}$, as shown in Fig. 8. Therefore, we confirmed that the influences, which evolved to mixed states, of dephasing coherent parameters in ${\rho }_{1}^{^{\prime}}$ and ${\rho }_{2}^{^{\prime}}$ (Eq. 18) can be reduced using a strong coherent state. Furthermore, by comparing with the dotted-blue box and dotted-red box in Fig. 8, the nonlinear optical gates (second and third) can yield high efficiencies (low error probabilities) and reliable performances (preserving pure quantum states) in the large amplitude range ($\alpha >100000$) of the coherent state.

According to our analysis, using the process model (Eq. 14), which can be used to formulate the interaction of XKNLs between a signal system (photon) and a probe beam (coherent state) via the master equation (Eq. 13), we can conclude that the only experimental condition is to utilize the strong coherent state (probe beam) to reduce the influences of photon loss and dephasing (decoherence effect). Hence, we can obtain high efficiencies (low error probabilities, Fig. 6) and reliable performances (values of coherent parameters approaching 1: Figs. 7 and 8) in the nonlinear optical gates. Consequently, the proposed procedure for generating single logical qubit information (quantum information on four-photon decoherence-free states) with immunity against collective decoherence can be experimentally implemented and is secure against photon loss and dephasing induced by the decoherence effect.

## Conclusion

We proposed a procedure (Fig. 2) that can generate four-photon decoherence-free states, logical qubits, and encode quantum information onto the superposition of logical qubits (single logical qubit information) using XKNLs and linear optical devices to secure quantum information against collective decoherence. In addition, in the nonlinear optical gates, first, second, third, fourth, and final gates, we analyzed the influences of photon loss and dephasing induced by the decoherence effect, and then demonstrated the experimental condition to obtain high efficiencies and reliable performances for the feasible procedure (Fig. 2). The advantages of our procedure are as follows:

1. Our procedure can be used to encode single logical qubit information and secure quantum information from collective decoherence using arbitrary information encoded onto the superposed state of four-photon decoherence-free states. The previous works, which proposed three-qubit decoherence-free states^37–41^, can provide the limited effect for the prevention against the affections of collective decoherence. Here, compared with three-qubit systems^37–41^, our scheme can generate four-qubit systems (four-photon decoherence-free states), which are more immune against collective decoherence, to maintain the coherence of quantum information.
2. In various quantum information processing schemes, including the procedure of generating decoherence-free states, the noises, photon loss and dephasing, induced by the decoherence effect is inevitable. Thus, the method to reduce the influences of decoherence effect should be proposed. Before the works^42,43^ using cavity-QED system for four-qubit decoherence-free states, their schemes overlooked the affections of decoherence effect. Here, in our scheme, we discussed the quantifications of the influences of photon loss and dephasing from the decoherence effect. Accordingly, we demonstrated the experimental condition by our analysis using master equation to utilize a strong, large amplitude, coherent state for the high efficiencies, low error probabilities, and reliable performances by preserving pure quantum state in nonlinear optical gates under the decoherence effect. Thus, our scheme is experimentally more feasible, compared with the previous works^42,43^.
3. In our procedure, the nonlinear optical gates utilized only the positive conditional phase shifts (${\uptheta }$) of the XKNLs with qubus beams and PNR measurements because it is generally not possible to change the sign of the conditional phase shift ($- {\uptheta }$) by Kok^76^. Moreover, the usage of a strong coherent state (for high efficiency and reliable performance) can yield an experimental advantage from using the weak conditional phase shift magnitude of the XKNL (i.e., if we use $\alpha$ = $10^{5}$ to reduce the influence of the decoherence effect, then the XKNL magnitude required is $\theta = 2.5 \times 10^{ - 5}$ for a fixed $\alpha \theta = 2.5$ and ${\text{P}}_{{{\text{err}}}} < 10^{ - 3}$). Hence, our nonlinear optical gates are more feasible in practice, as realizing a large conditional phase shift magnitude via the XKNL is difficult (extremely weak: $\theta \approx 10^{ - 18}$)^77–79^.

Consequently, our procedure, which can generate four-photon decoherence-free states, logical qubits, and encode quantum information onto the superposition of logical qubits (single logical qubit information), was designed to prevent quantum information from collective decoherence. In addition, for the feasible procedure (Fig. 2), we demonstrated that the nonlinear optical gates (first, second, third, fourth, and final) can yield high efficiencies, obtaining low error probabilities, and reliable performances, preserving pure quantum state, against photon loss and dephasing of the decoherence effect when a strong coherent state, probe beam, was used.
